# Supplementary figures and images for: MCPH1 inhibits Condensin II during interphase by regulating its SMC2-Kleisin interface
Source: eLife. 2021 Dec 1;10:e73348. doi: 10.7554/eLife.73348 (PMC8673838; doi:10.7554/eLife.73348)

Figure 1 A - Source data 1

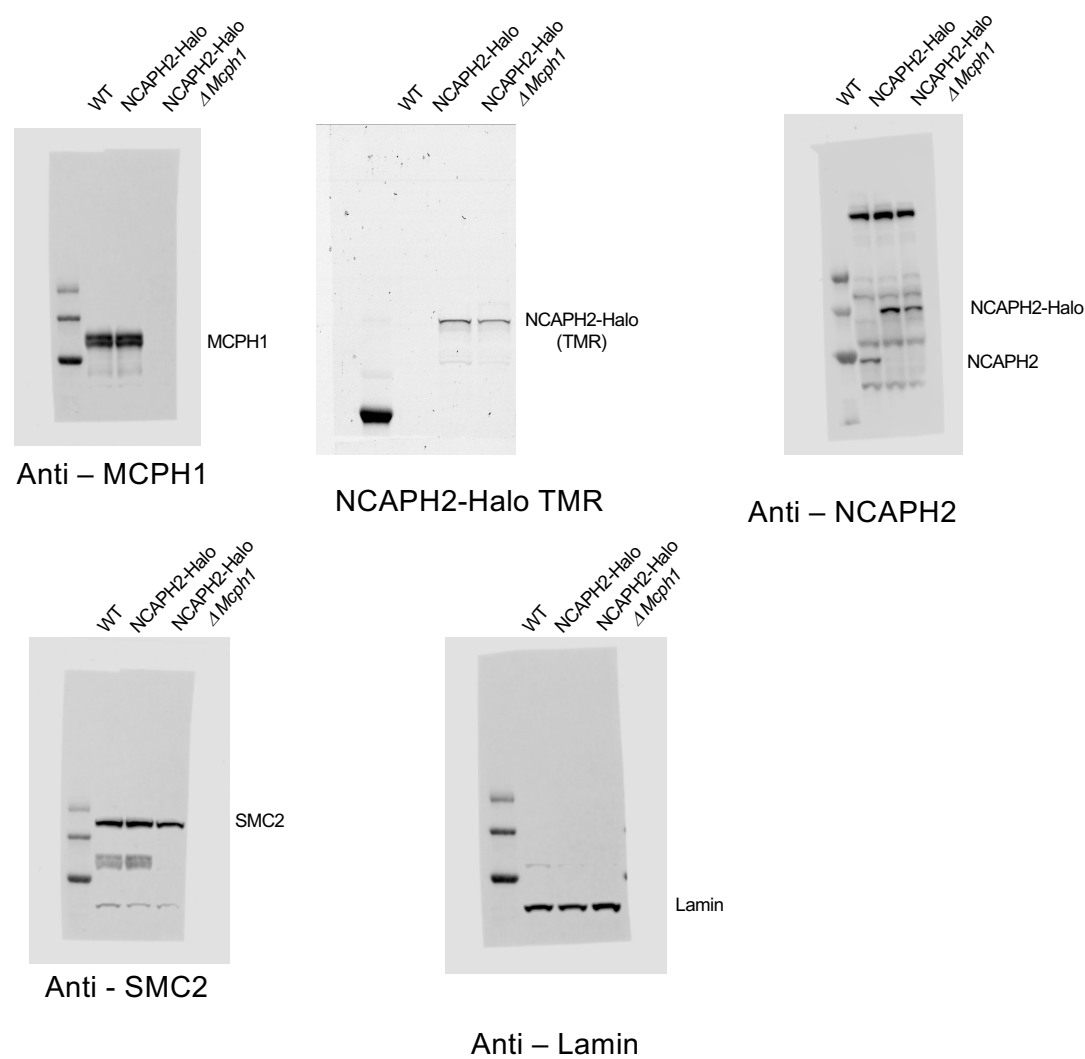

Supplement: Figure 1—source data 1. [file elife-73348-fig1-data1.zip › Figure 1-source data 1 /Figure 1-source data 1.pdf]

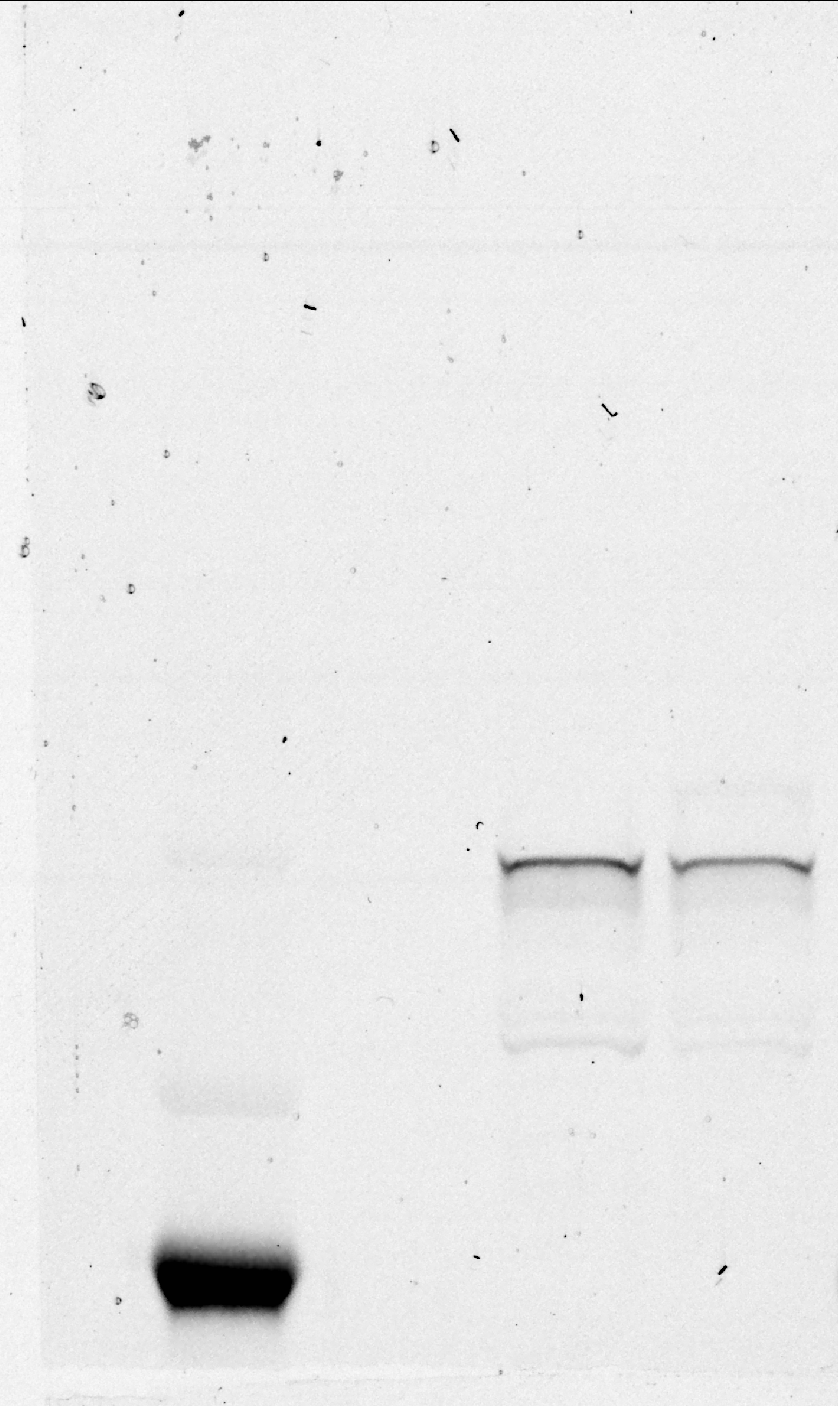

Supplement: Figure 1—source data 1. [file elife-73348-fig1-data1.zip › Figure 1-source data 1 /Figure 1-source data 1-Halo TMR.tiff]

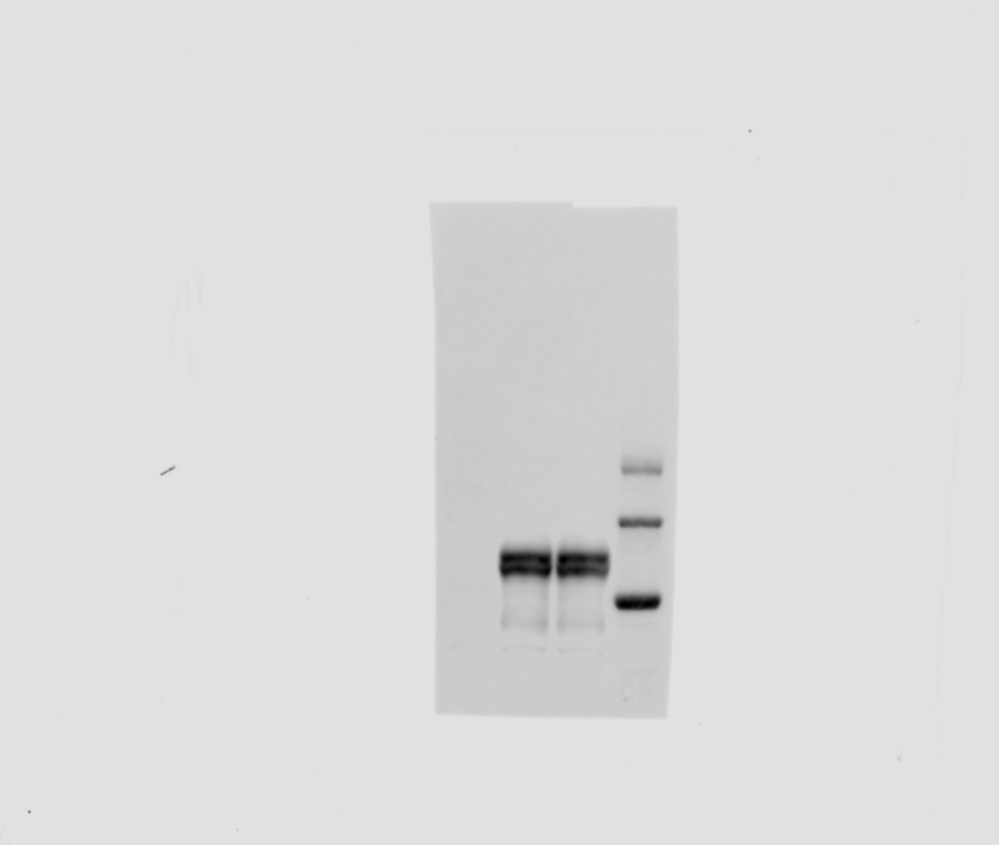

Supplement: Figure 1—source data 1. [file elife-73348-fig1-data1.zip › Figure 1-source data 1 /Figure 1-source data 1-MCPH1.jpg]

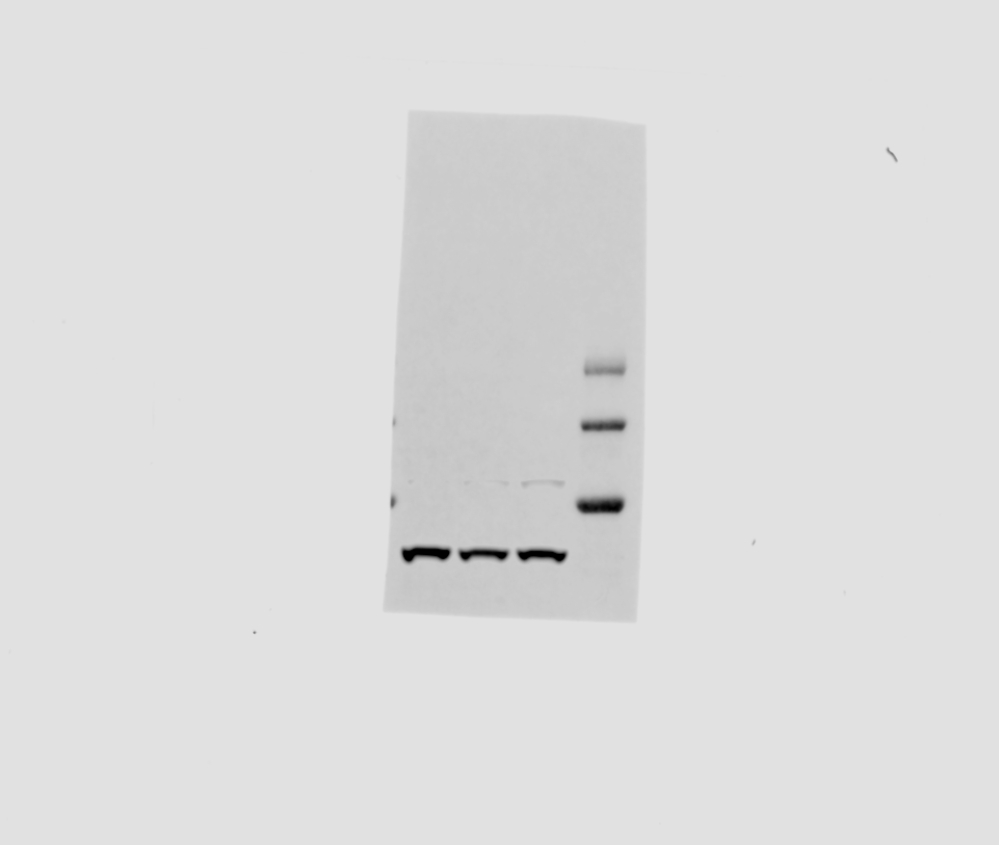

Supplement: Figure 1—source data 1. [file elife-73348-fig1-data1.zip › Figure 1-source data 1 /Figure 1-source data 1-Lamin.jpg]

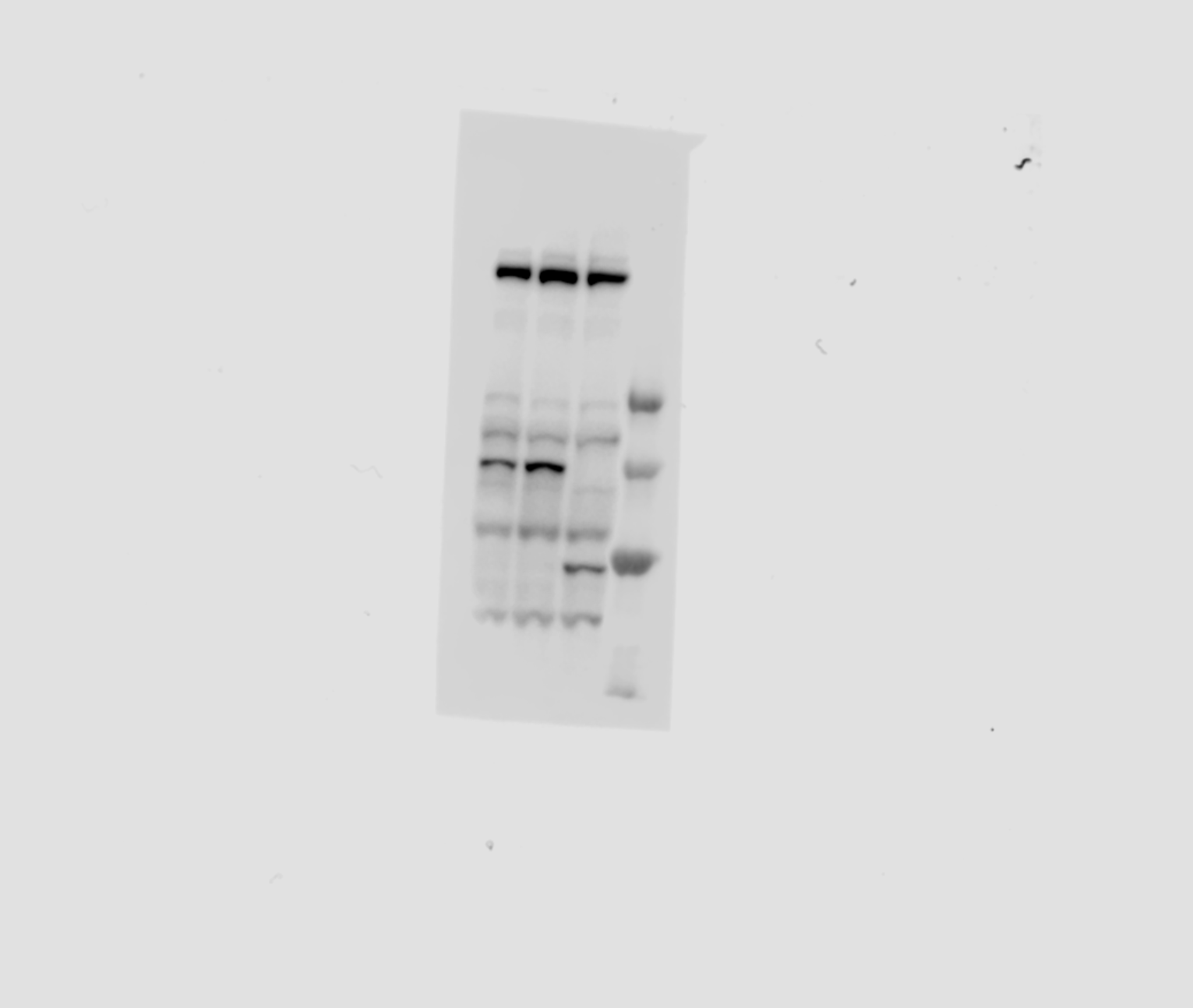

Supplement: Figure 1—source data 1. [file elife-73348-fig1-data1.zip › Figure 1-source data 1 /Figure 1-source data 1-NCAPH2.tif]

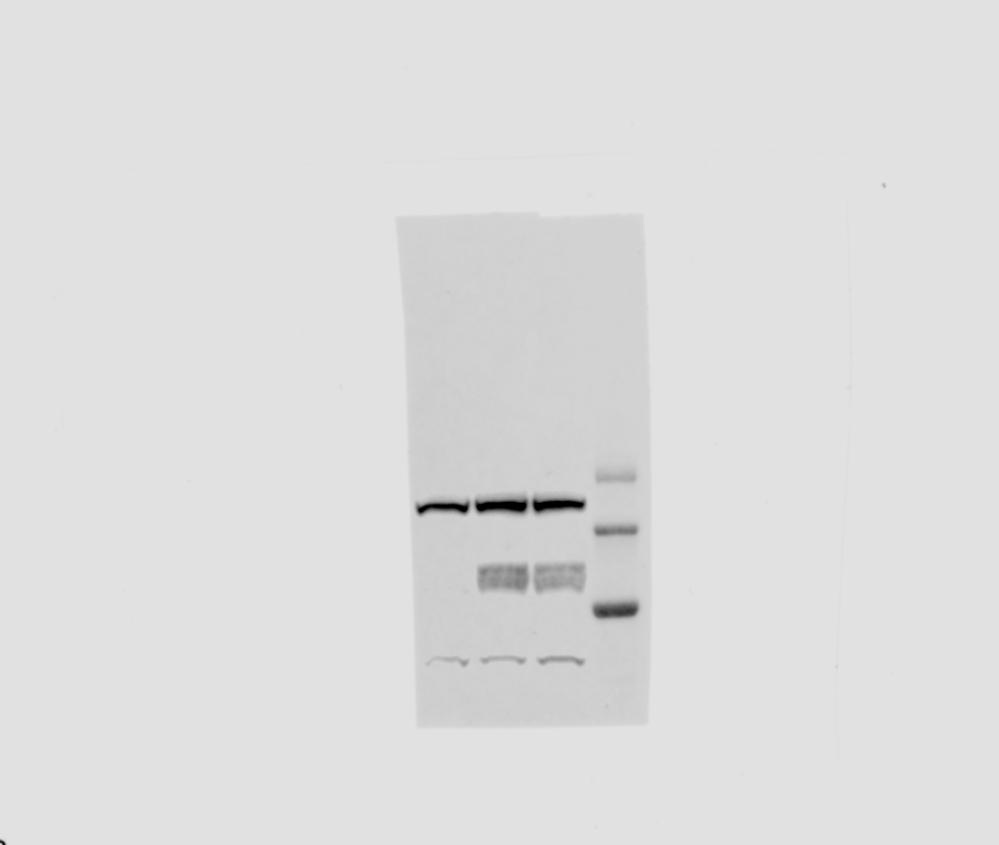

Supplement: Figure 1—source data 1. [file elife-73348-fig1-data1.zip › Figure 1-source data 1 /Figure 1-source data 1-SMC2.jpg]

Figure 1 D - Source data 1

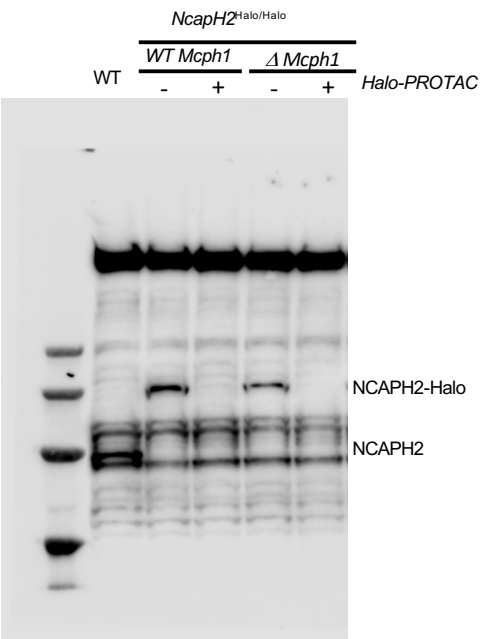

Anti – NCAPH2

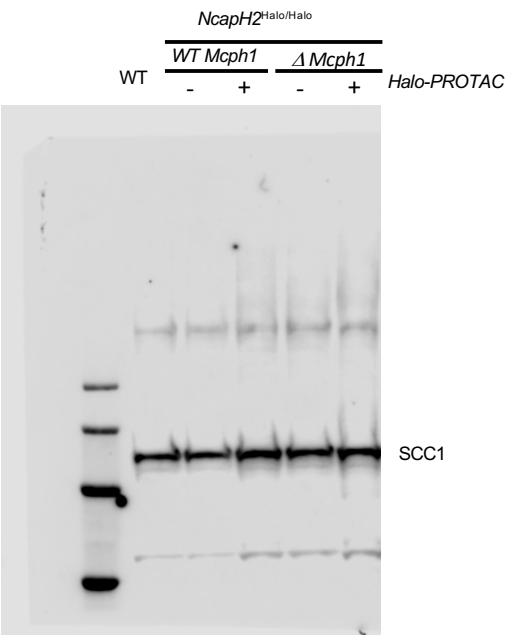

Anti – SCC1

Supplement: Figure 1—source data 2. [file elife-73348-fig1-data2.zip › Figure 1-source data 2/Figure 1-source data 2.pdf]

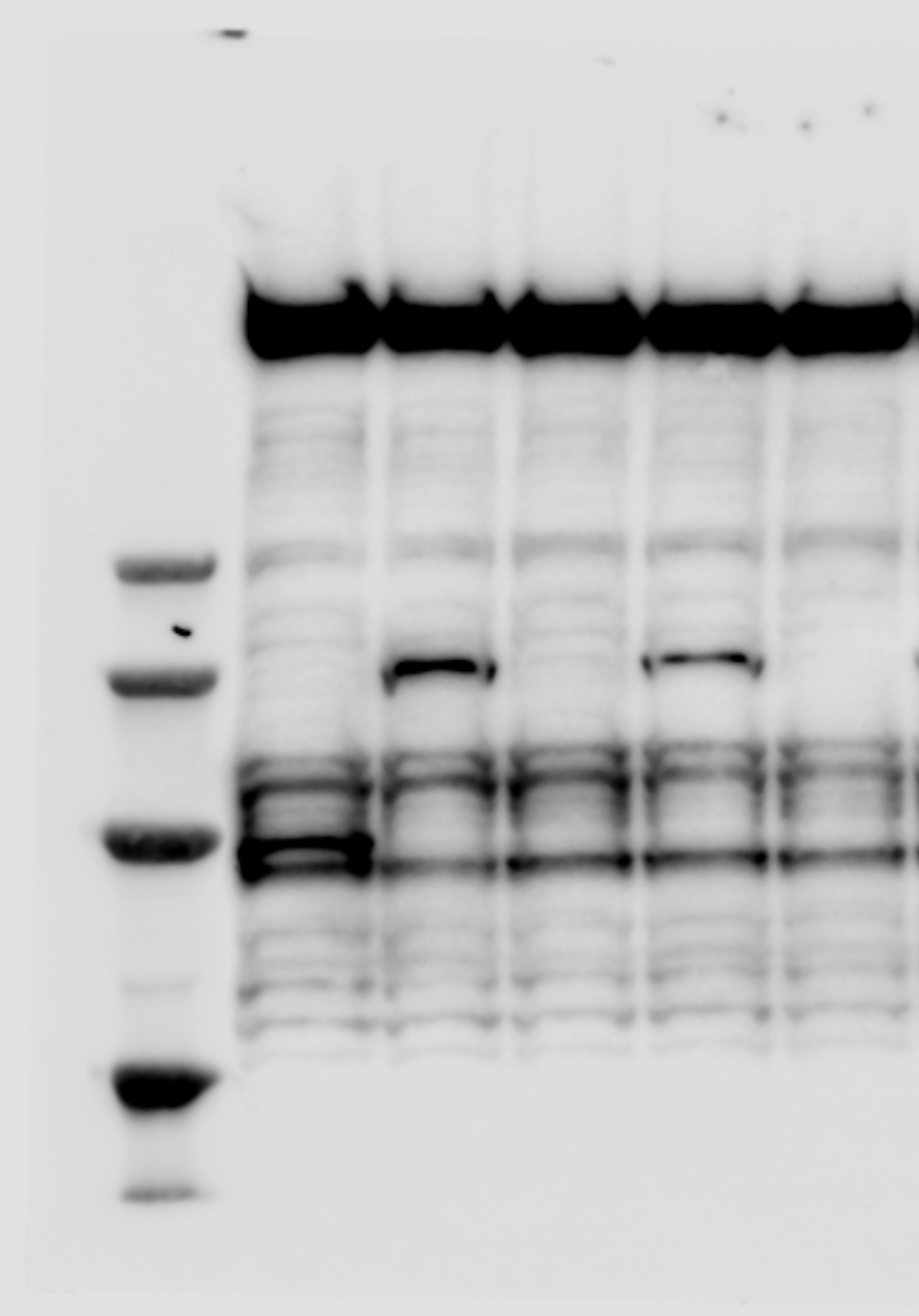

Supplement: Figure 1—source data 2. [file elife-73348-fig1-data2.zip › Figure 1-source data 2/Figure 1-source data 2-NCAPH2.tiff]

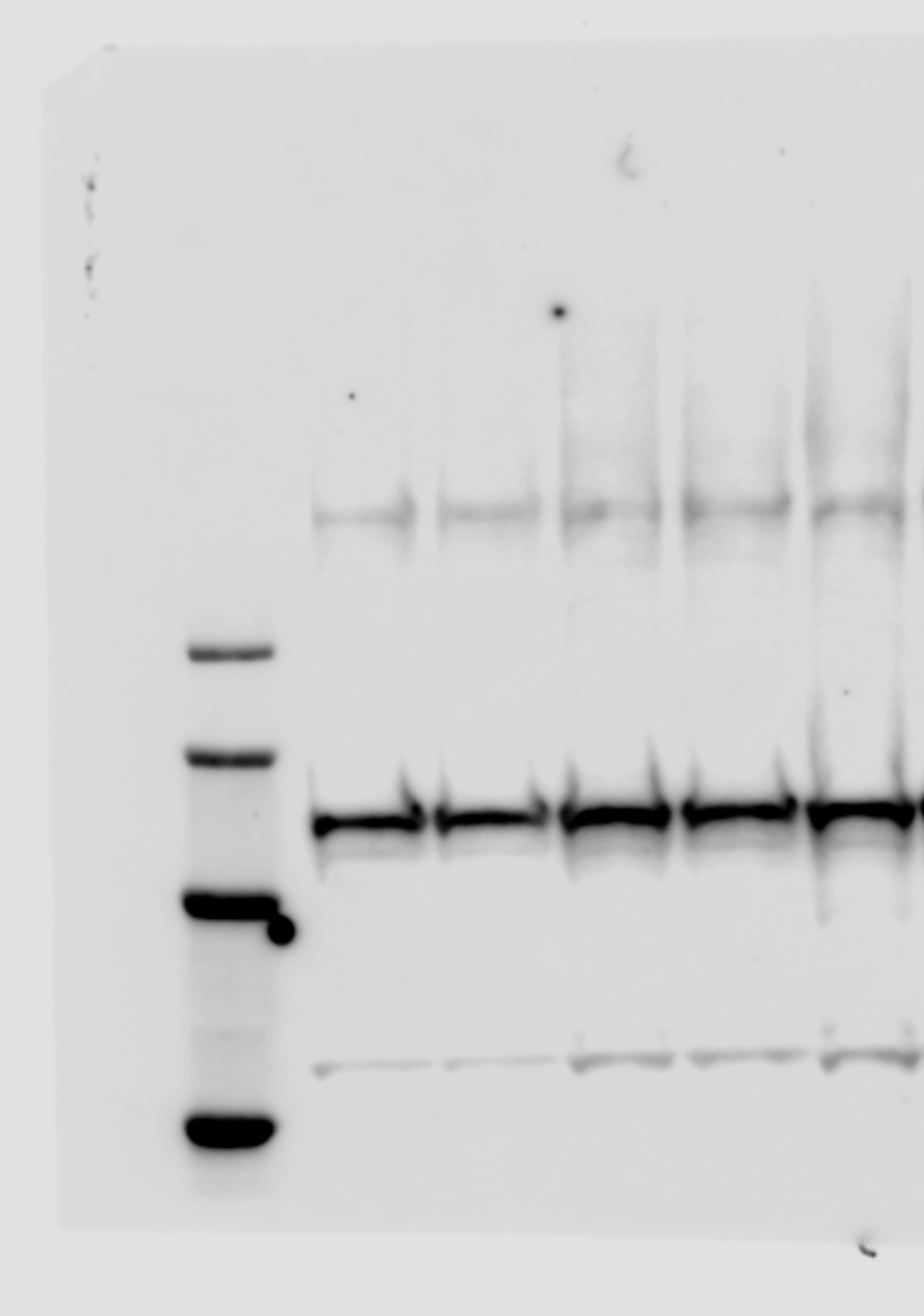

Supplement: Figure 1—source data 2. [file elife-73348-fig1-data2.zip › Figure 1-source data 2/Figure 1-source data 2-SCC1.tiff]

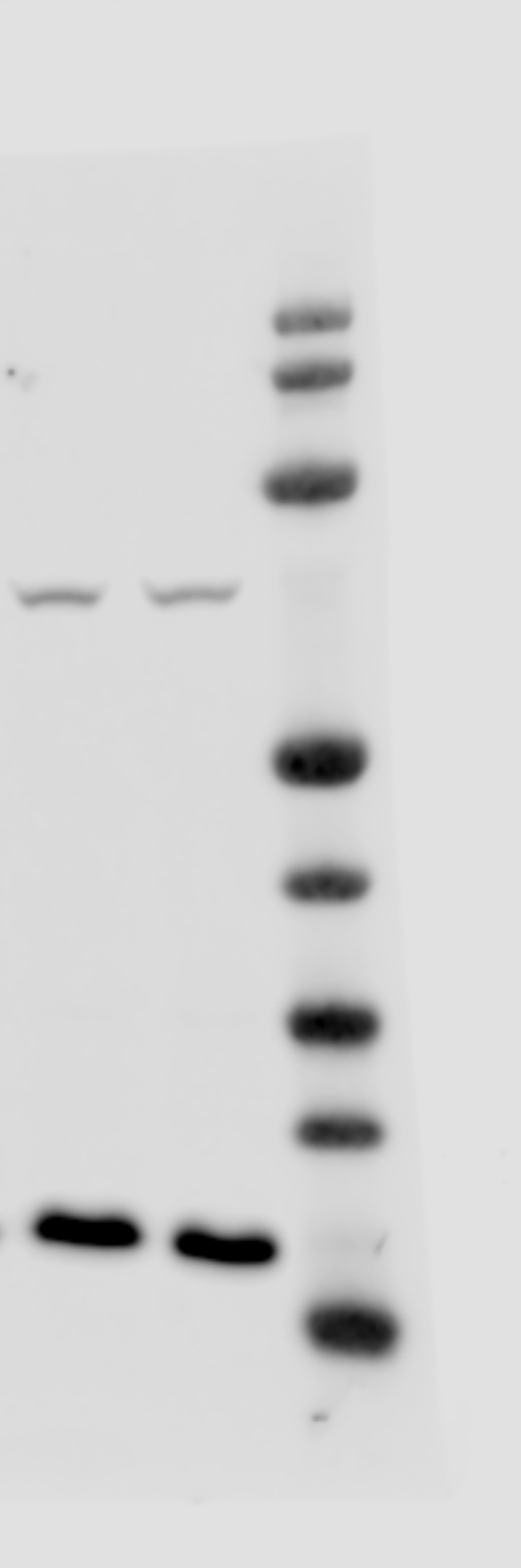

Supplement: Figure 1—figure supplement 1—source data 1. [file elife-73348-fig1-figsupp1-data1.zip › Figure 1-figure supplement 1-source data 1/Figure 1ΓÇôfigure supplement 1-source data 1-anti gH2AX.tiff]

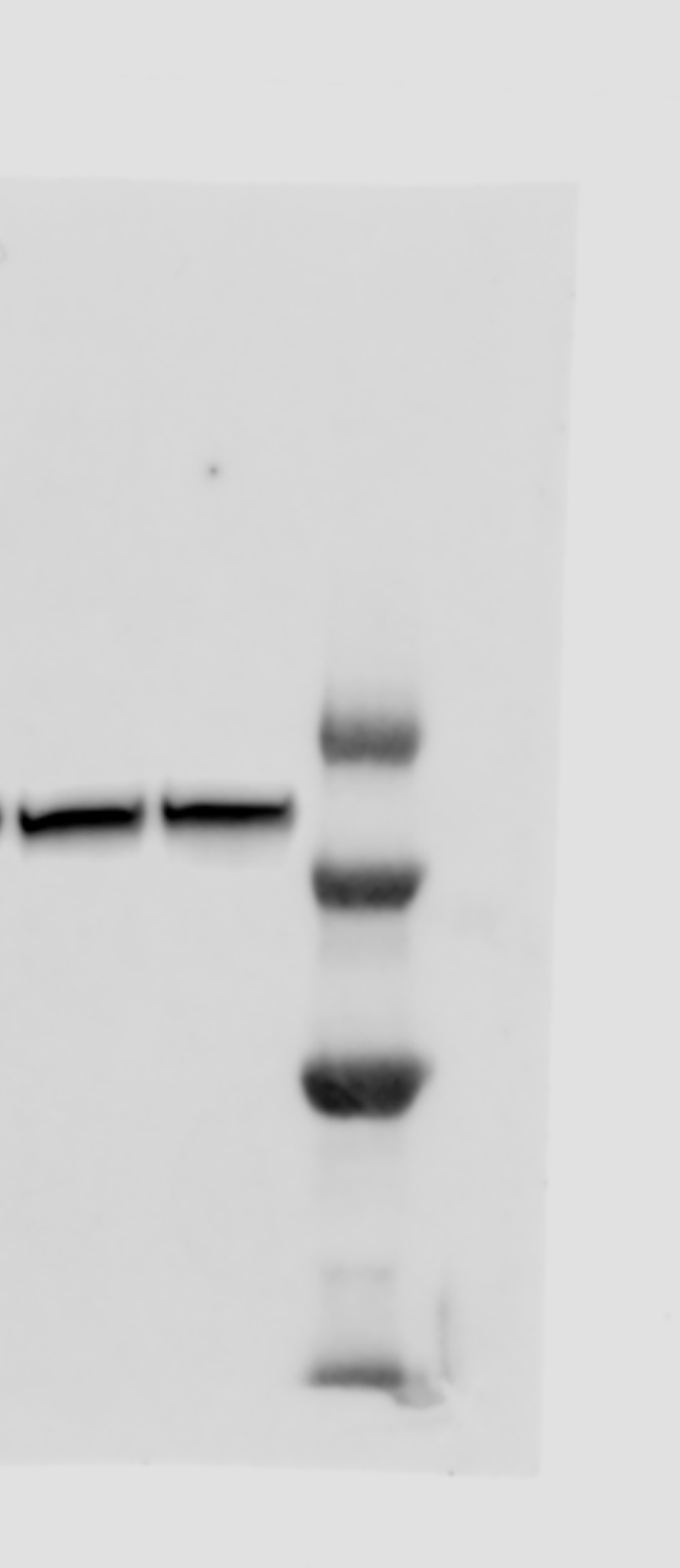

Supplement: Figure 1—figure supplement 1—source data 1. [file elife-73348-fig1-figsupp1-data1.zip › Figure 1-figure supplement 1-source data 1/Figure 1ΓÇôfigure supplement 1-source data 1-anti SMC2.tiff]

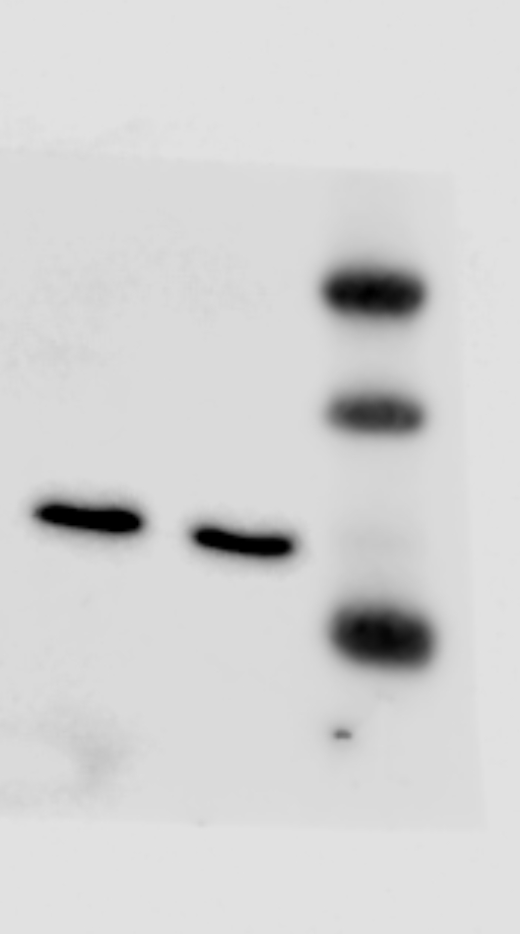

Supplement: Figure 1—figure supplement 1—source data 1. [file elife-73348-fig1-figsupp1-data1.zip › Figure 1-figure supplement 1-source data 1/Figure 1ΓÇôfigure supplement 1-source data 1-antiH3PS10.tiff]

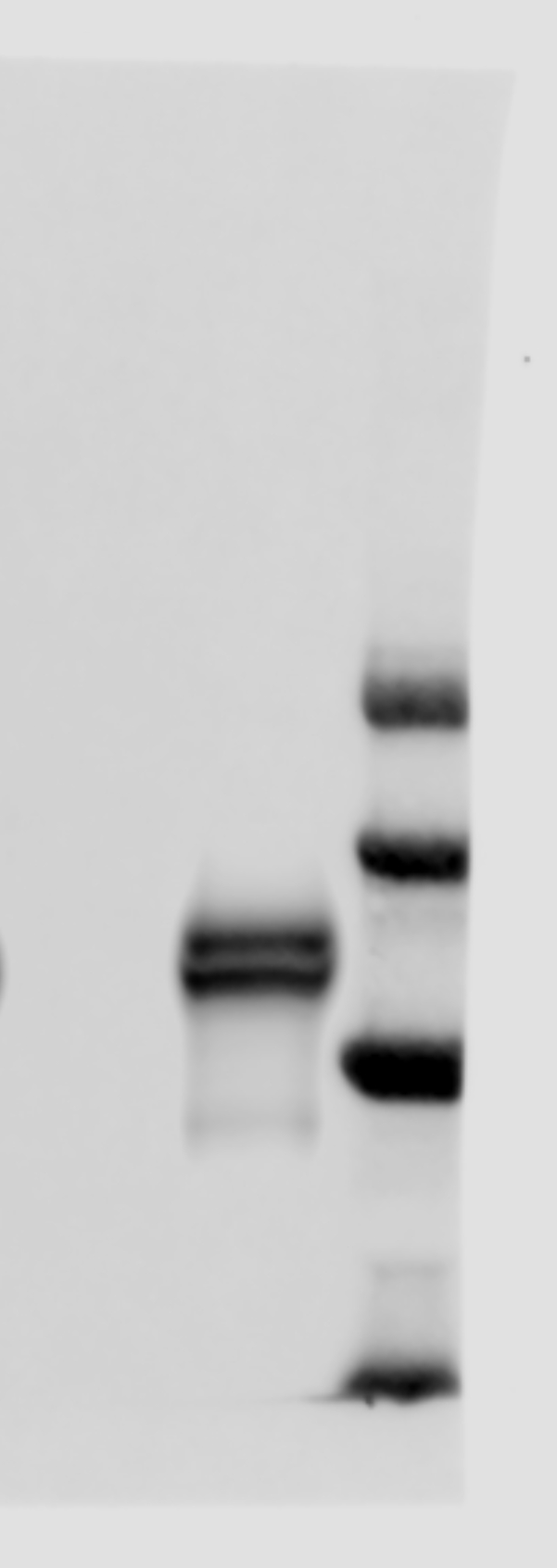

Supplement: Figure 1—figure supplement 1—source data 1. [file elife-73348-fig1-figsupp1-data1.zip › Figure 1-figure supplement 1-source data 1/Figure 1ΓÇôfigure supplement 1-source data 1-anti MCPH1.tiff]

Figure 1 – figure supplement 1

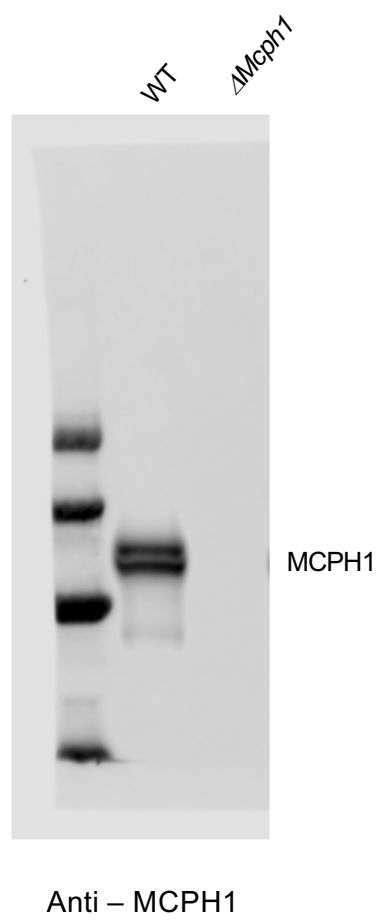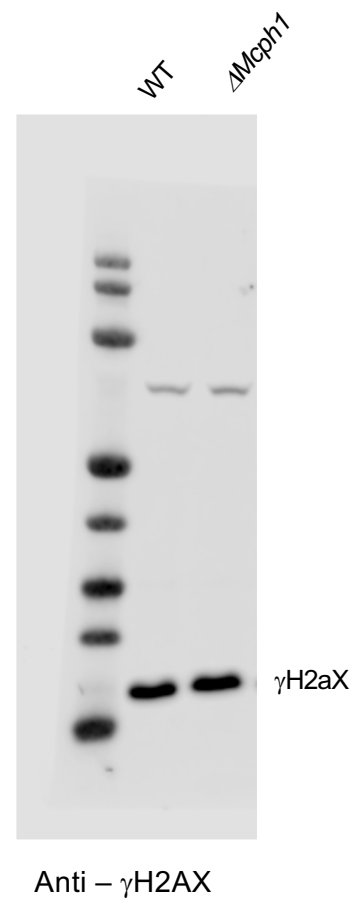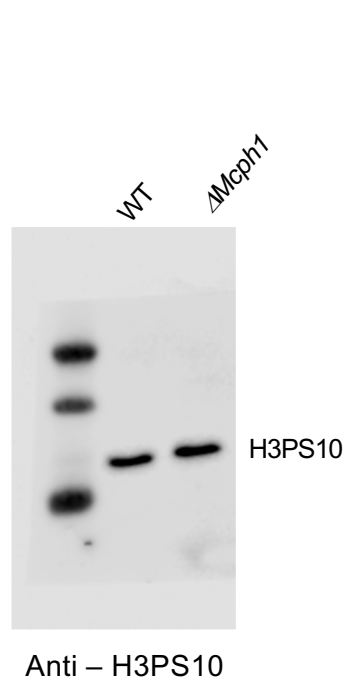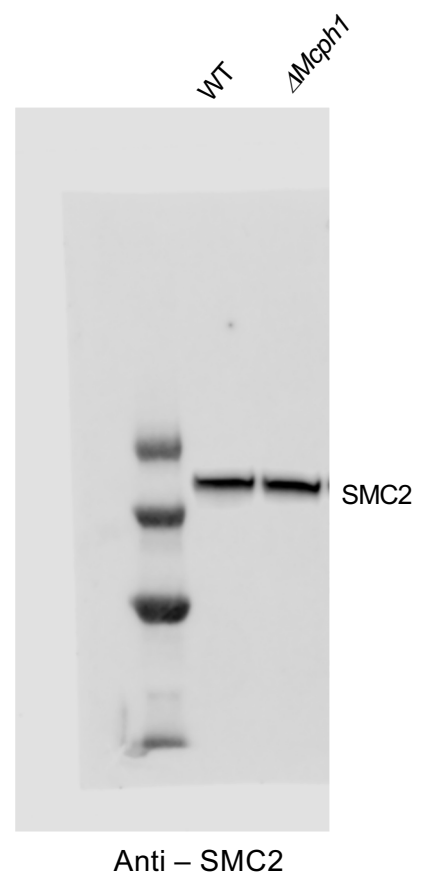

Supplement: Figure 1—figure supplement 1—source data 1. [file elife-73348-fig1-figsupp1-data1.zip › Figure 1-figure supplement 1-source data 1/Figure 1ΓÇôfigure supplement 1-source data 1.pdf]

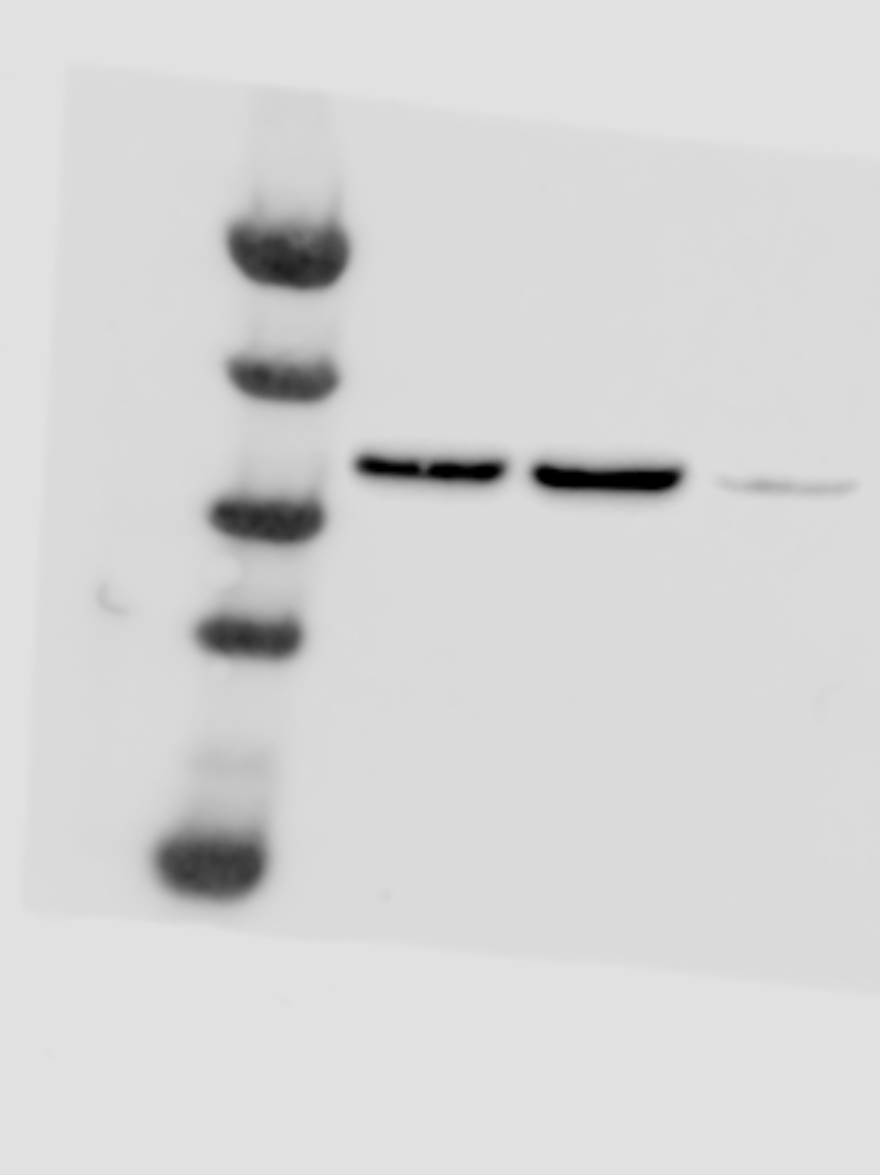

Supplement: Figure 3—source data 1. [file elife-73348-fig3-data1.zip › Figure 3-source data 1 /Figure 3A-source data 1-anti-phospho CDK1.tiff]

Figure 3 A - Source data 1

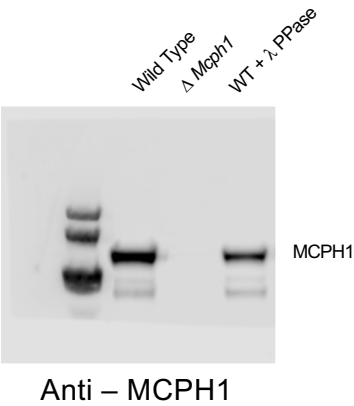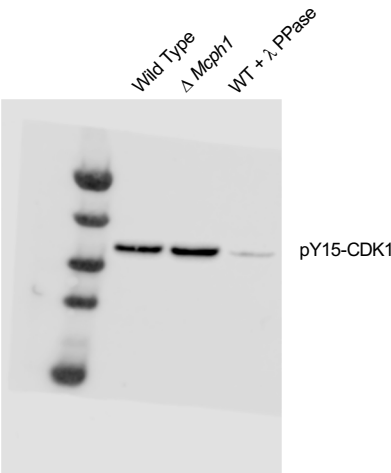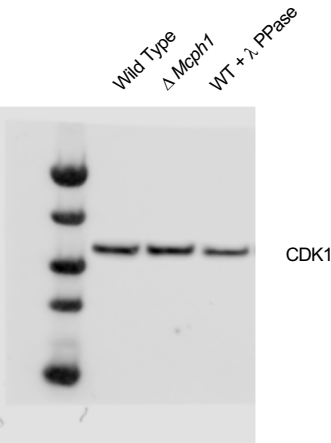

Supplement: Figure 3—source data 1. [file elife-73348-fig3-data1.zip › Figure 3-source data 1 /Figure 3A-source data 1.pdf]

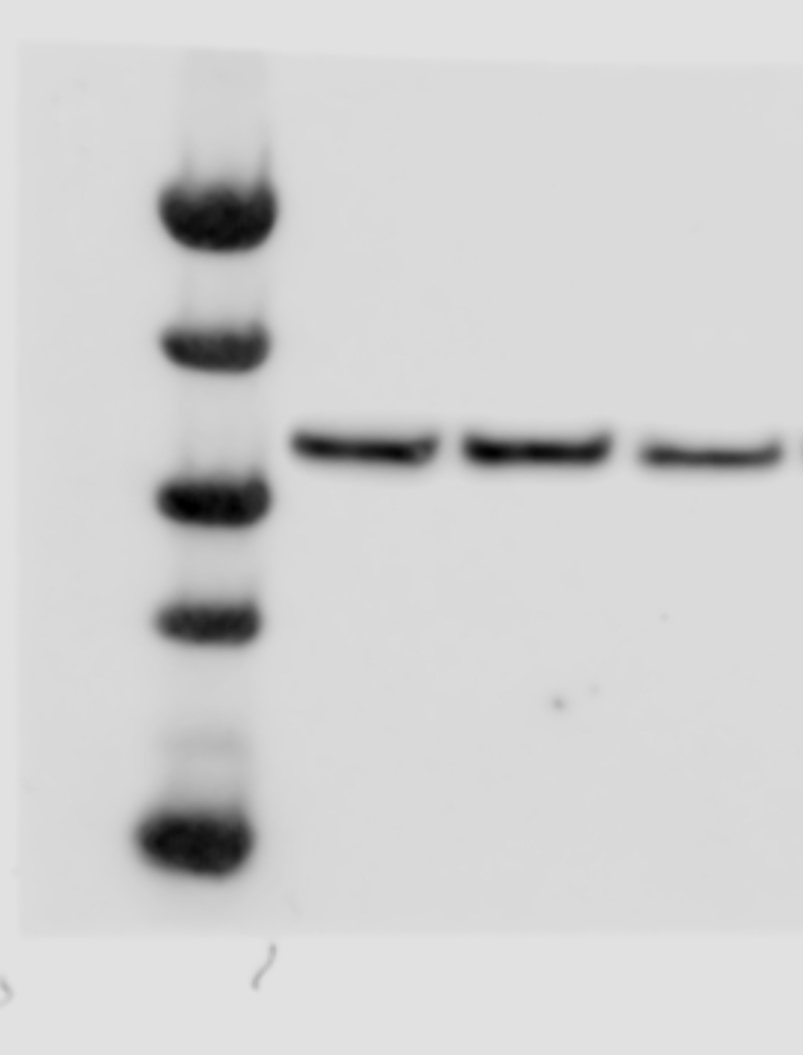

Supplement: Figure 3—source data 1. [file elife-73348-fig3-data1.zip › Figure 3-source data 1 /Figure 3A-source data 1-anti-CDK1.jpg]

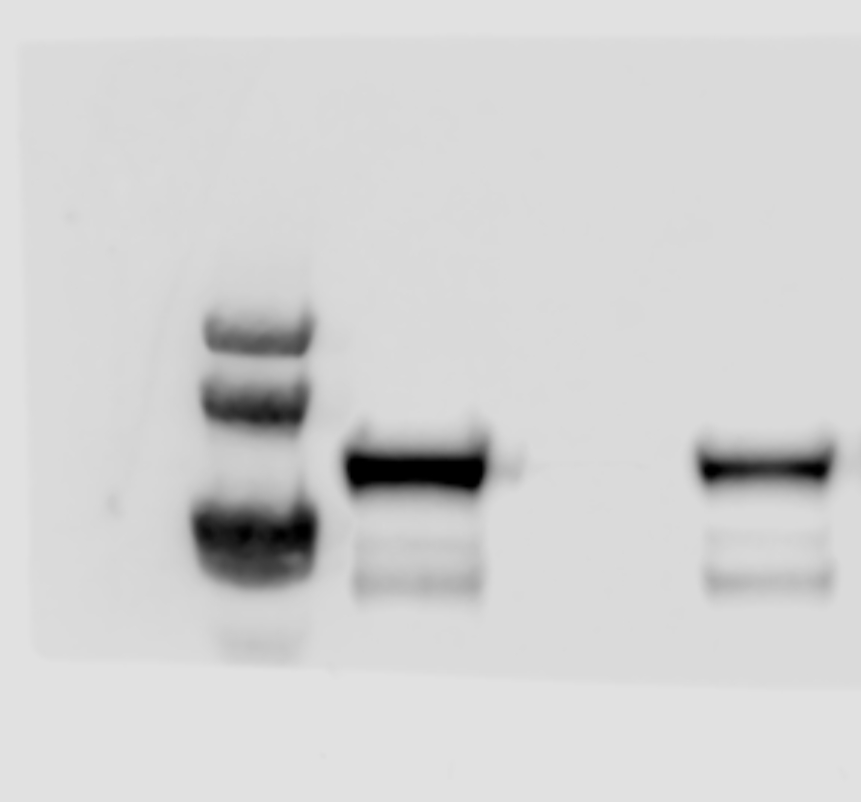

Supplement: Figure 3—source data 1. [file elife-73348-fig3-data1.zip › Figure 3-source data 1 /Figure 3A-source data 1-anti MCPH1.tiff]

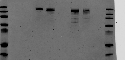

Supplement: Figure 5—source data 3. [file elife-73348-fig5-data3.zip › Figure 5_source data 3/Figure 5D-source data 1.jpg]

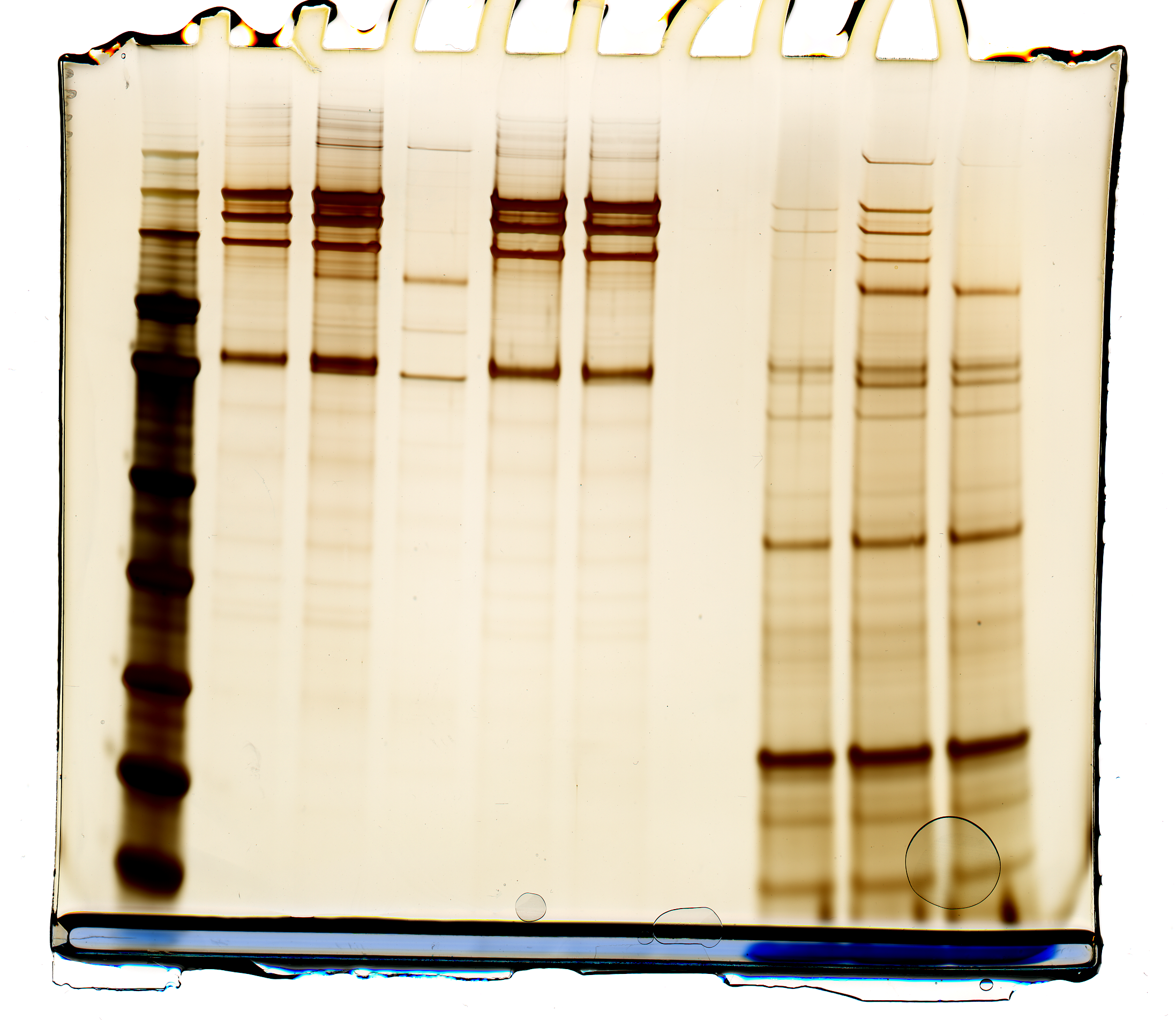

Supplement: Figure 5—source data 3. [file elife-73348-fig5-data3.zip › Figure 5_source data 3/Figure 5C-source data 1.tif]

Figure 5 – figure source data 1

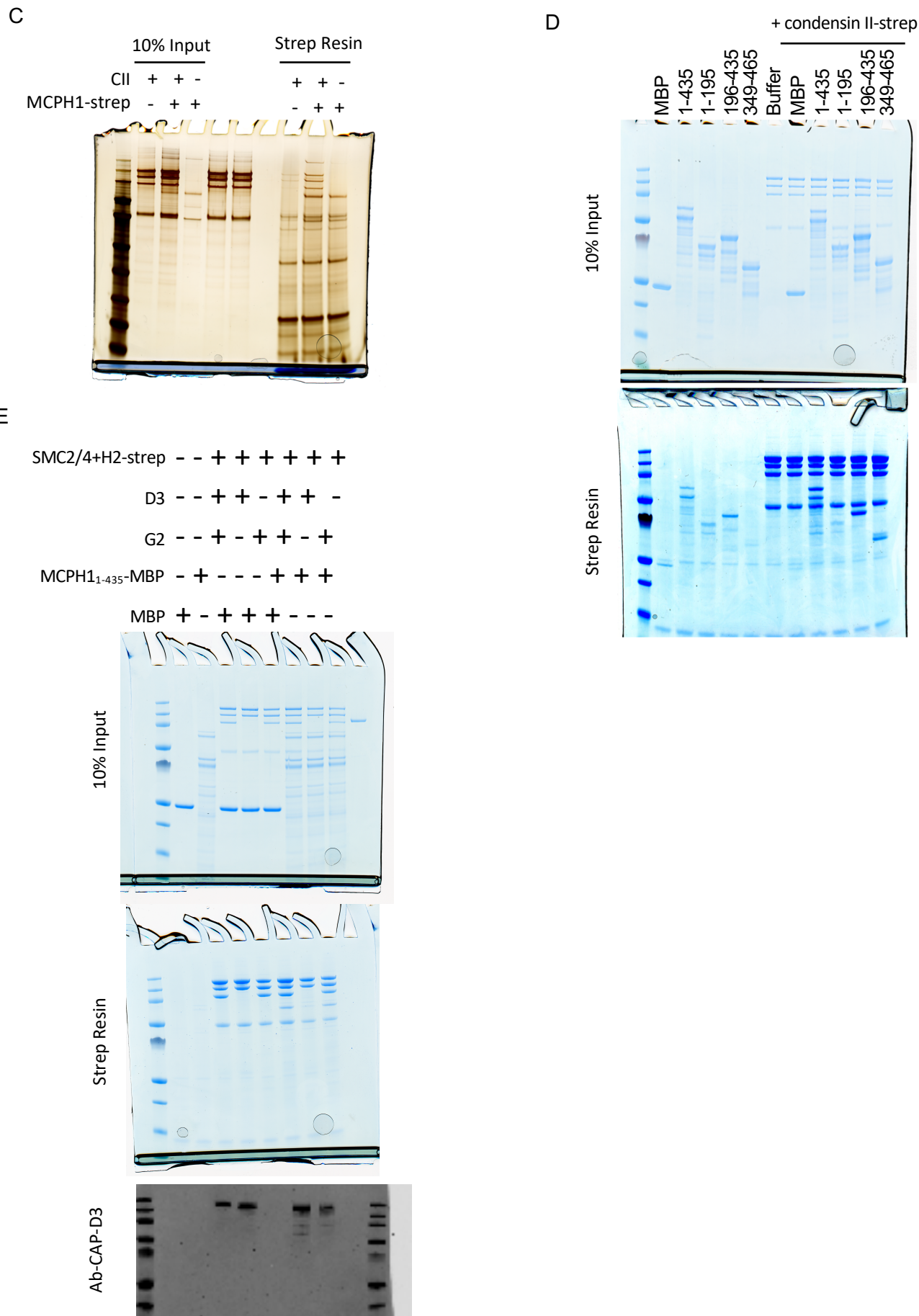

Supplement: Figure 5—source data 3. [file elife-73348-fig5-data3.zip › Figure 5_source data 3/Figure 5-source data 1.pdf]

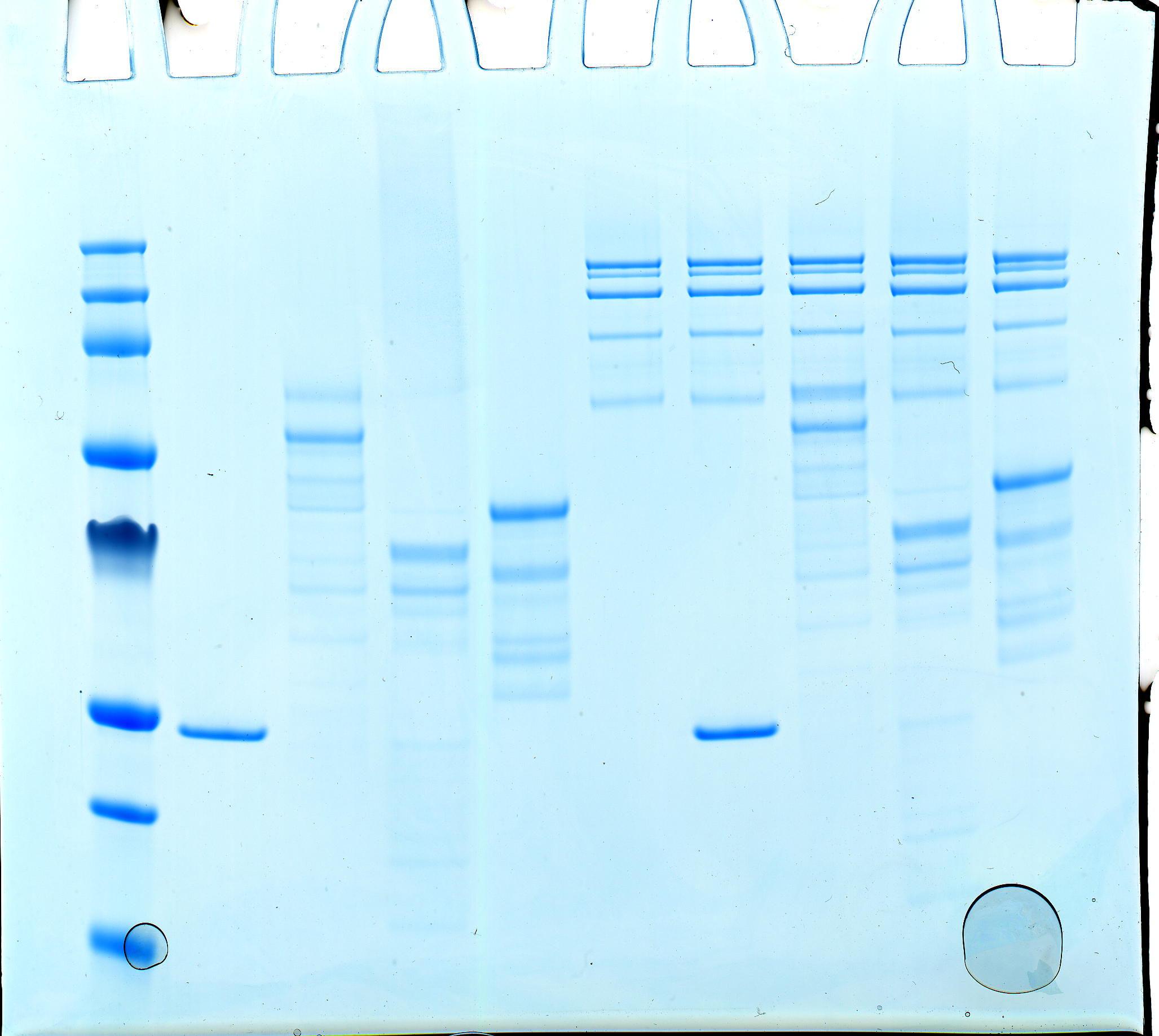

Supplement: Figure 5—figure supplement 1—source data 1. [file elife-73348-fig5-figsupp1-data1.zip › Figure 5-figure supplement 1-source data 1/Figure 5 -figure supplement 1A-source data 1.tif]

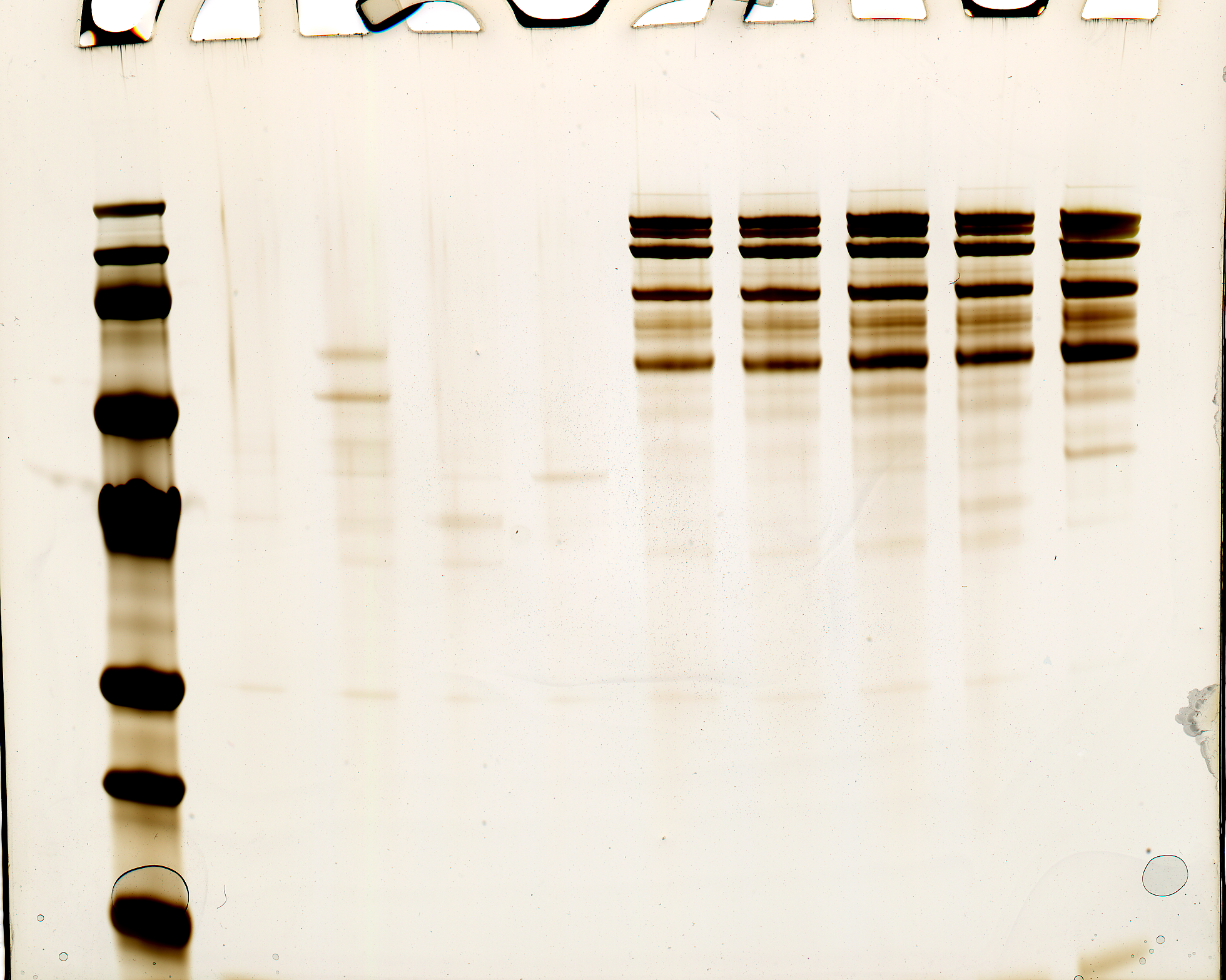

Supplement: Figure 5—figure supplement 1—source data 1. [file elife-73348-fig5-figsupp1-data1.zip › Figure 5-figure supplement 1-source data 1/Figure 5 -figure supplement 1A-source data 2.tif]

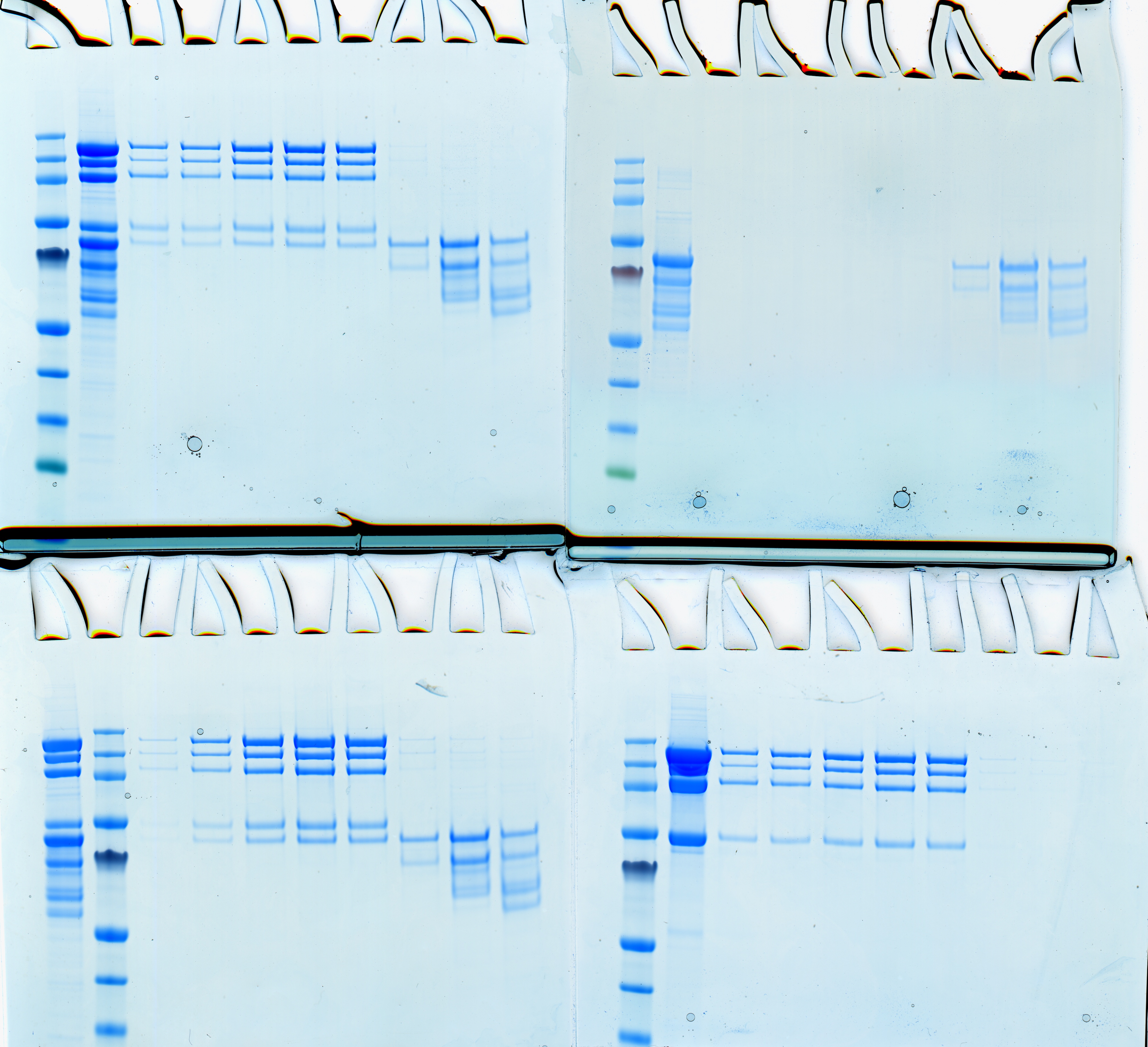

Supplement: Figure 5—figure supplement 1—source data 1. [file elife-73348-fig5-figsupp1-data1.zip › Figure 5-figure supplement 1-source data 1/Figure 5 -figure supplement 1C-source data 1.jpg]

Figure 5 – figure supplement 1-figure source data 1

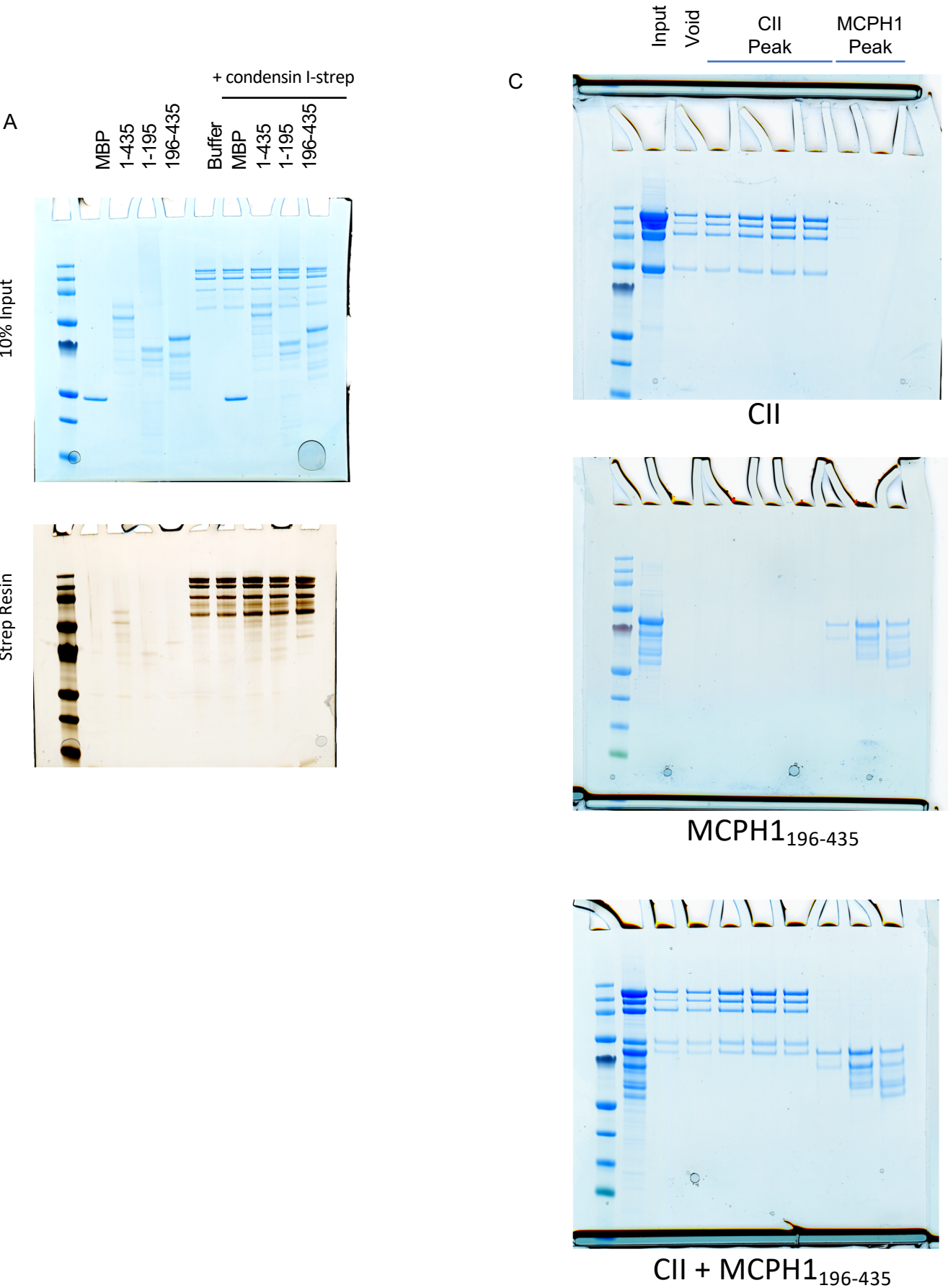

Supplement: Figure 5—figure supplement 1—source data 1. [file elife-73348-fig5-figsupp1-data1.zip › Figure 5-figure supplement 1-source data 1/Figure 5-figure supplement 1_figure source data.pdf]

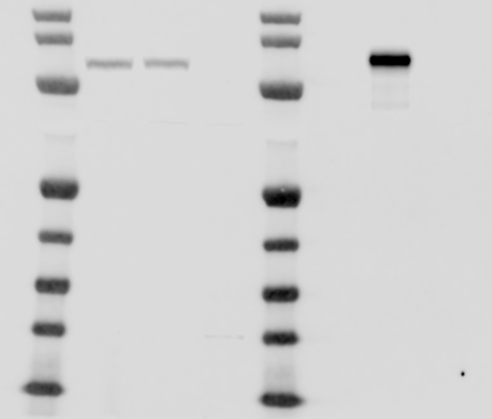

Supplement: Figure 6—source data 1. [file elife-73348-fig6-data1.zip › Figure 6-source data 1/Figure 6A-source data 1-anti MCPH1.tif]

Figure 6 A - Source data 1

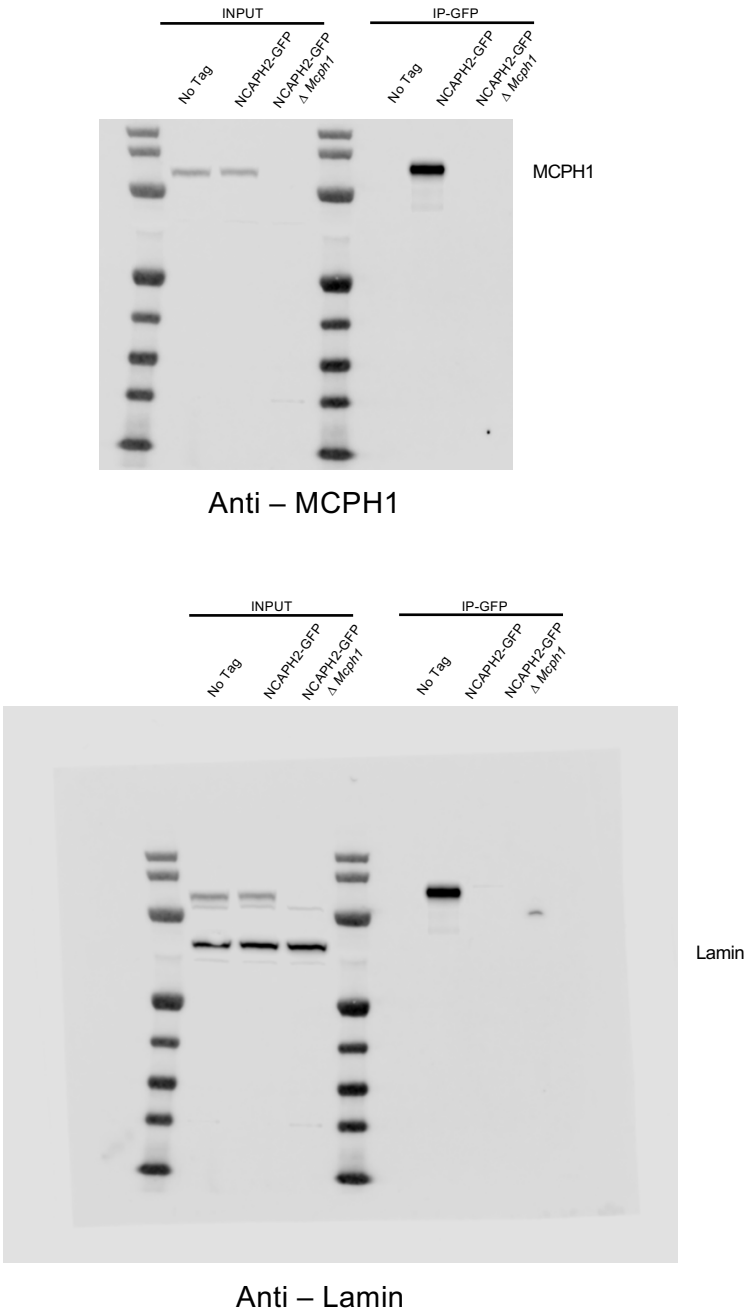

Supplement: Figure 6—source data 1. [file elife-73348-fig6-data1.zip › Figure 6-source data 1/Figure 6A-source data 1.pdf]

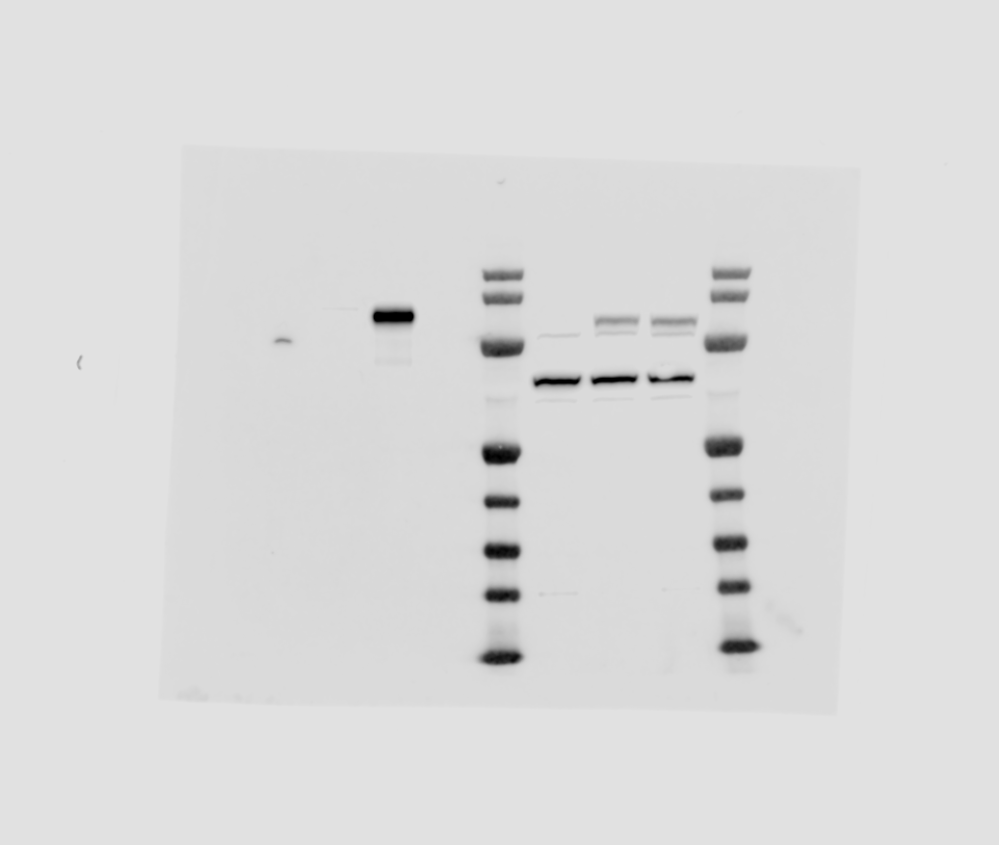

Supplement: Figure 6—source data 1. [file elife-73348-fig6-data1.zip › Figure 6-source data 1/Figure 6A-source data 1-anti Lamin.tif]

Figure 6 B - Source data 1

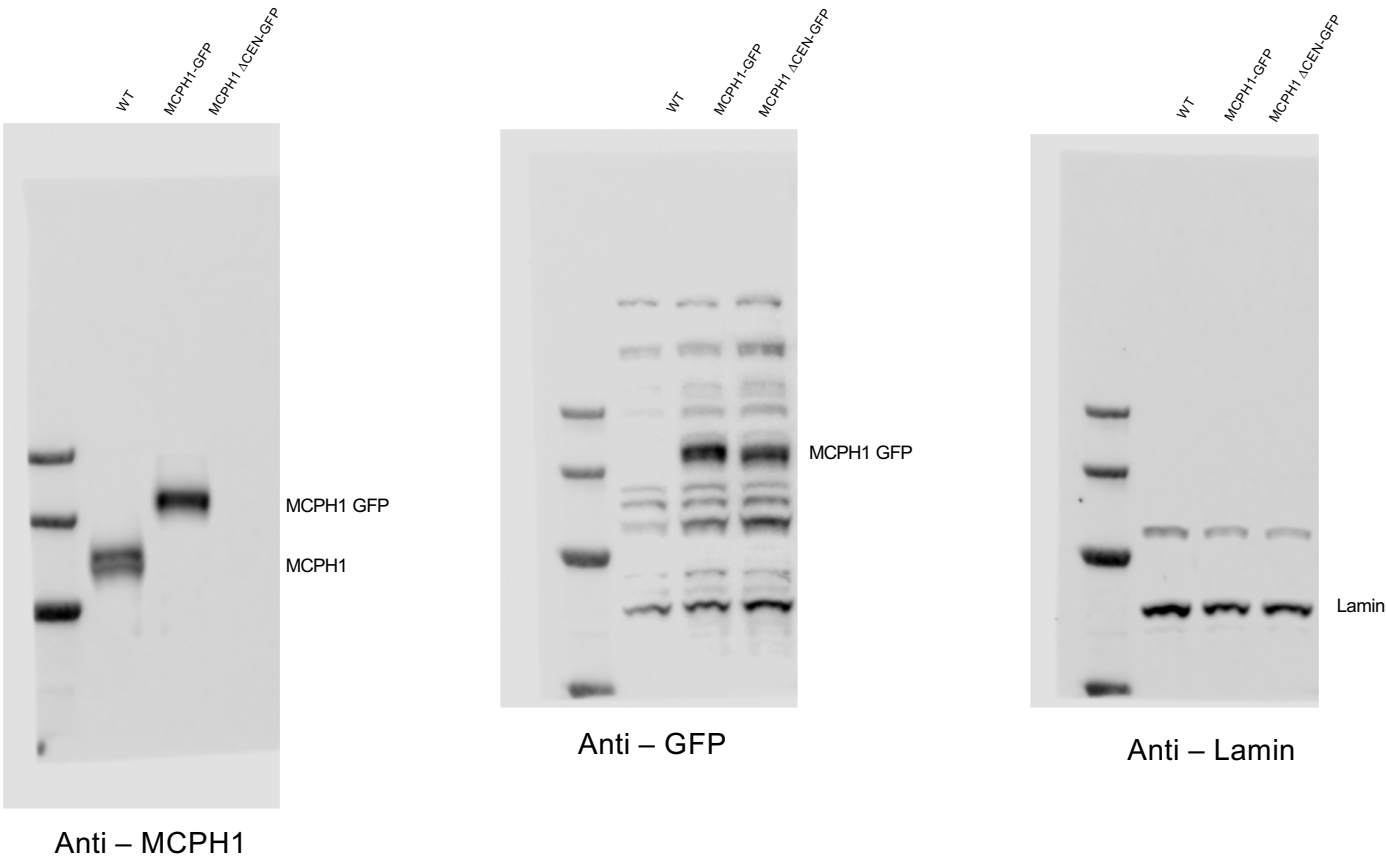

Supplement: Figure 6—source data 2. [file elife-73348-fig6-data2.zip › Figure 6-source data 2/Figure 6B-source data 1.pdf]

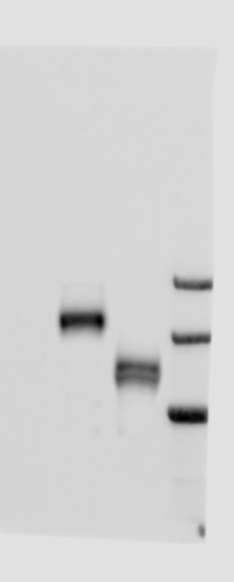

Supplement: Figure 6—source data 2. [file elife-73348-fig6-data2.zip › Figure 6-source data 2/Figure 6B-source data 1-anti MCPH1.tiff]

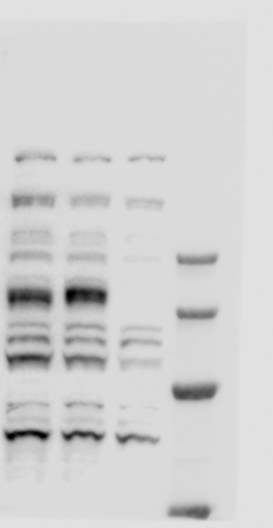

Supplement: Figure 6—source data 2. [file elife-73348-fig6-data2.zip › Figure 6-source data 2/Figure 6B-source data 1-anti GFP.tiff]

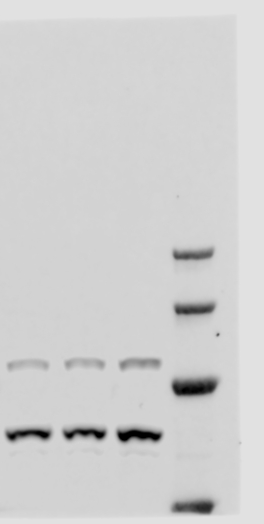

Supplement: Figure 6—source data 2. [file elife-73348-fig6-data2.zip › Figure 6-source data 2/Figure 6B-source data 1-anti Lamin.tiff]

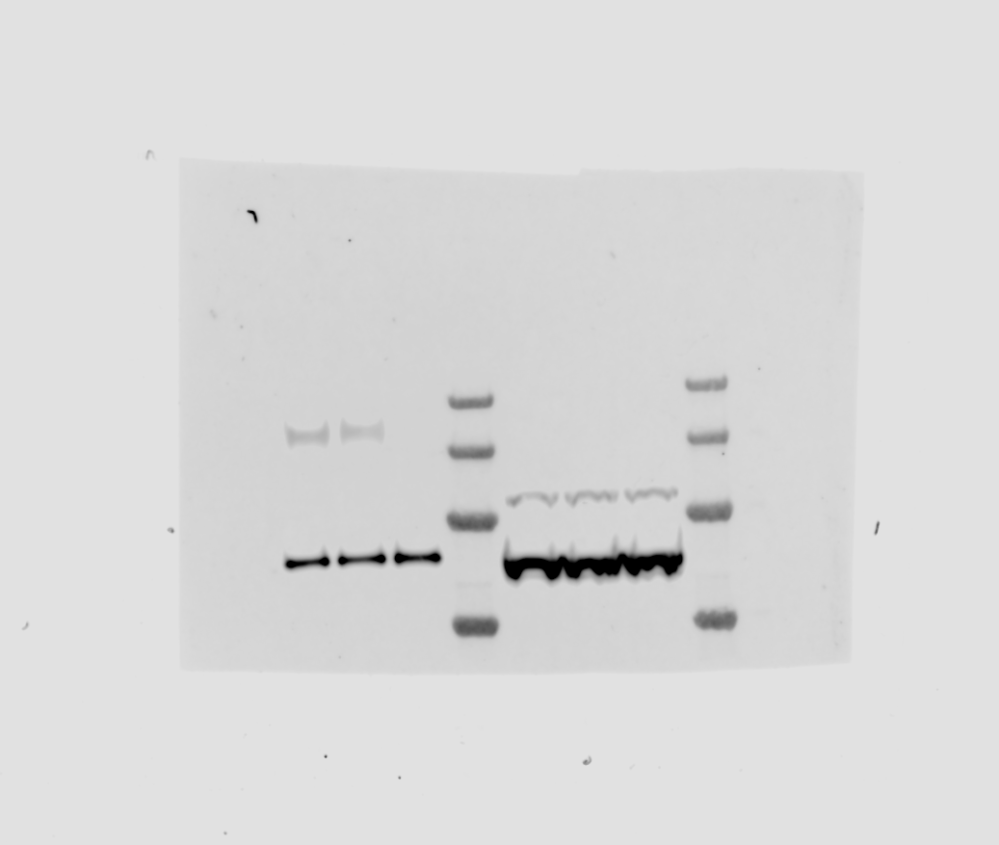

Supplement: Figure 6—source data 3. [file elife-73348-fig6-data3.zip › Figure 6-source data 3/Figure 6C-source data 1-anti Lamin.tif]

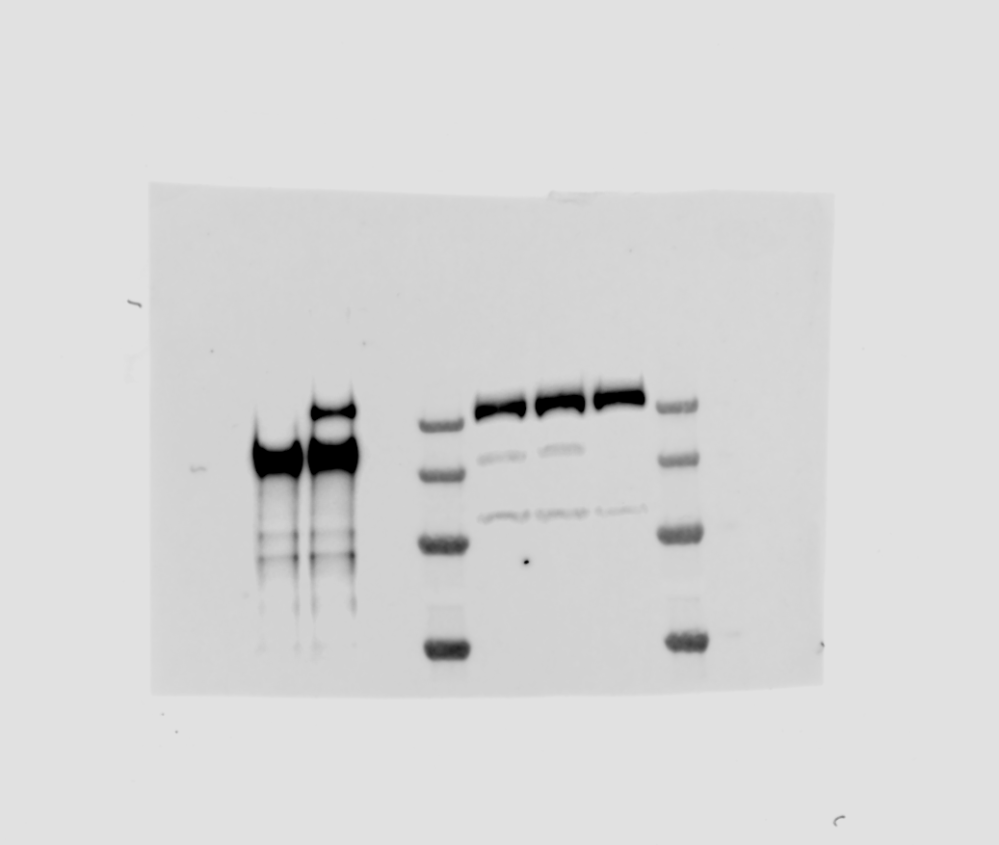

Supplement: Figure 6—source data 3. [file elife-73348-fig6-data3.zip › Figure 6-source data 3/Figure 6C-source data 1-anti CapD3.tif]

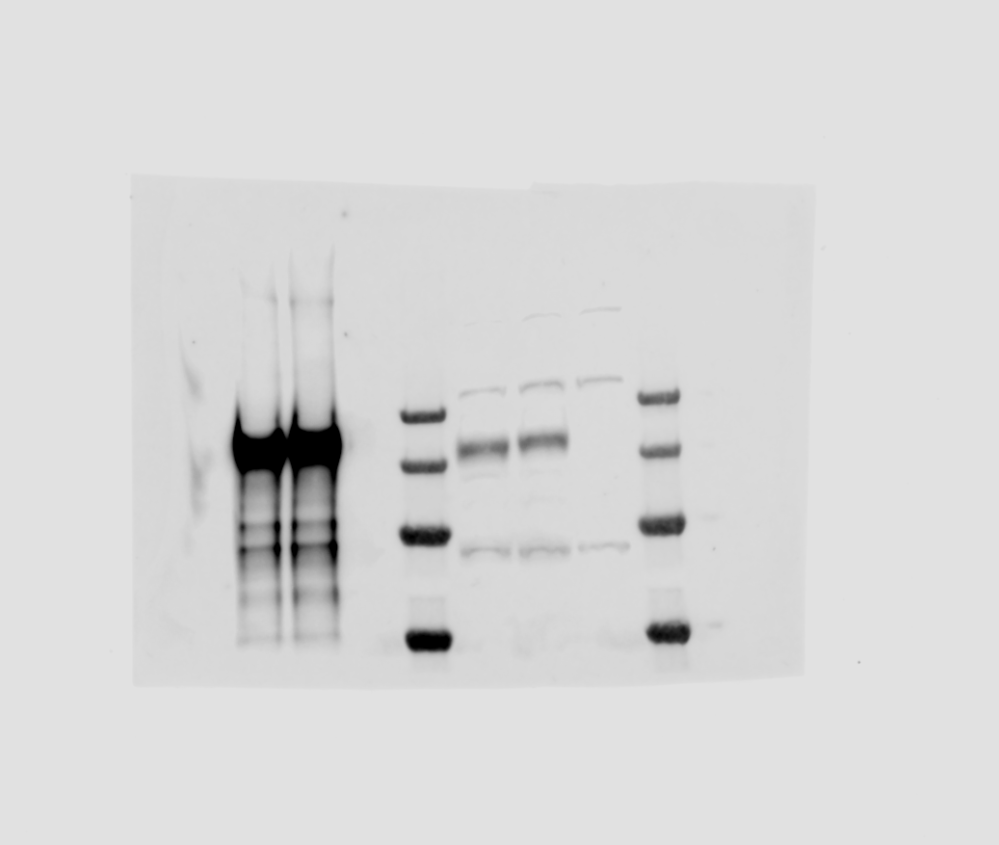

Supplement: Figure 6—source data 3. [file elife-73348-fig6-data3.zip › Figure 6-source data 3/Figure 6C-source data 1-anti GFP.png]

Figure 6 C - Source data 1

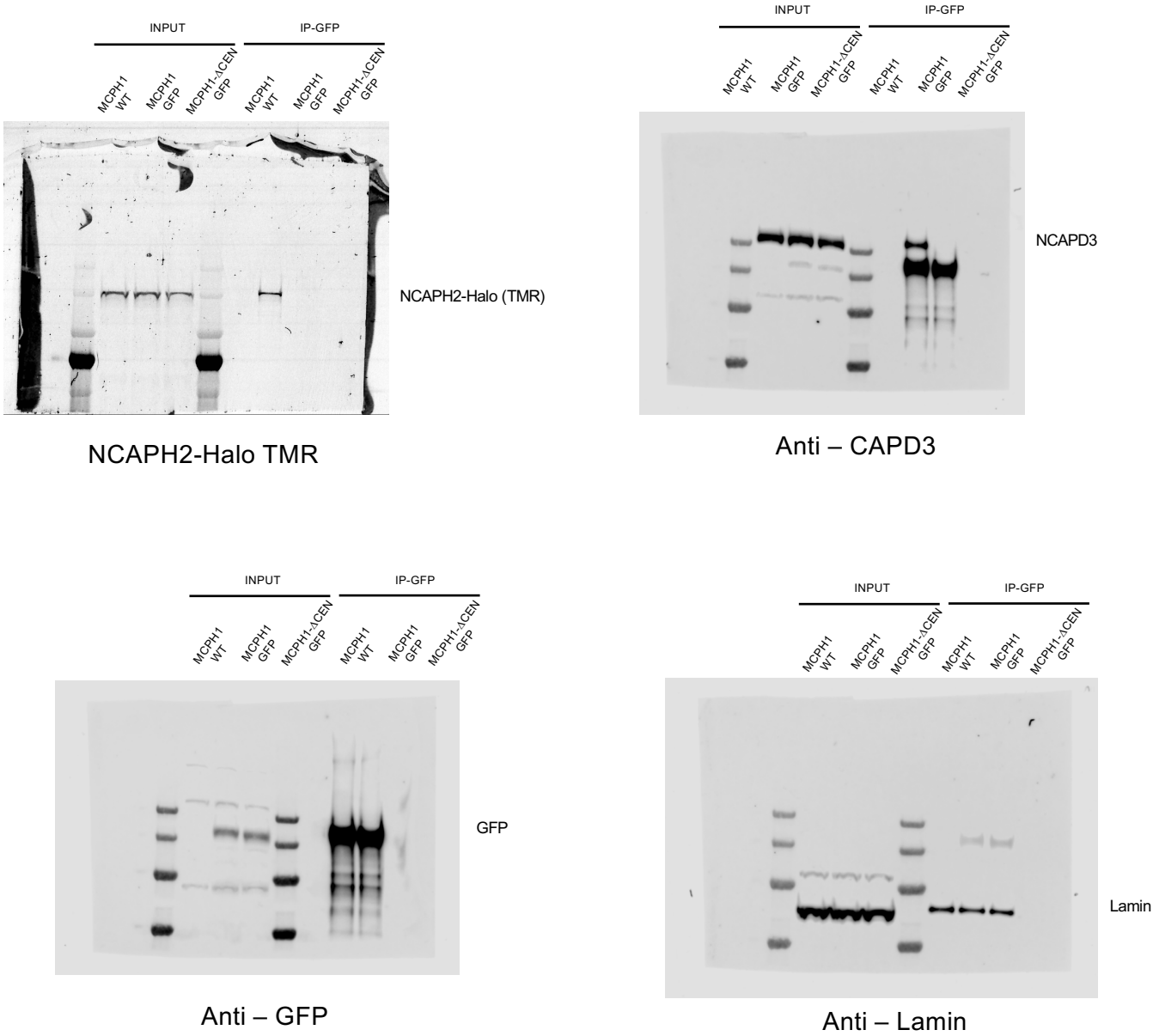

Supplement: Figure 6—source data 3. [file elife-73348-fig6-data3.zip › Figure 6-source data 3/Figure 6C-source data 1.pdf]

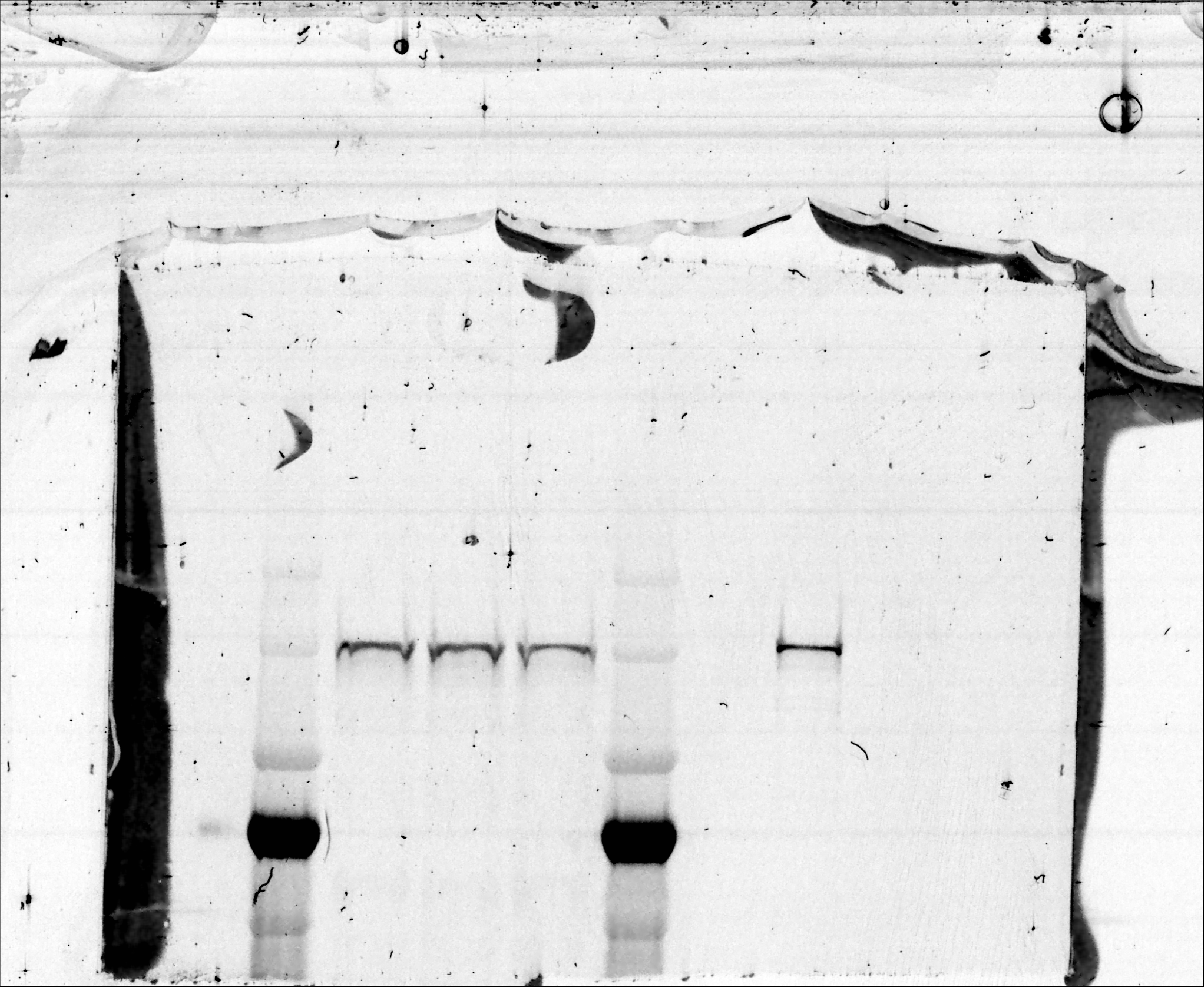

Supplement: Figure 6—source data 3. [file elife-73348-fig6-data3.zip › Figure 6-source data 3/Figure 6C-source data 1-NCAPH2-Halo TMR.tif]

Figure 7 -figure source data 1

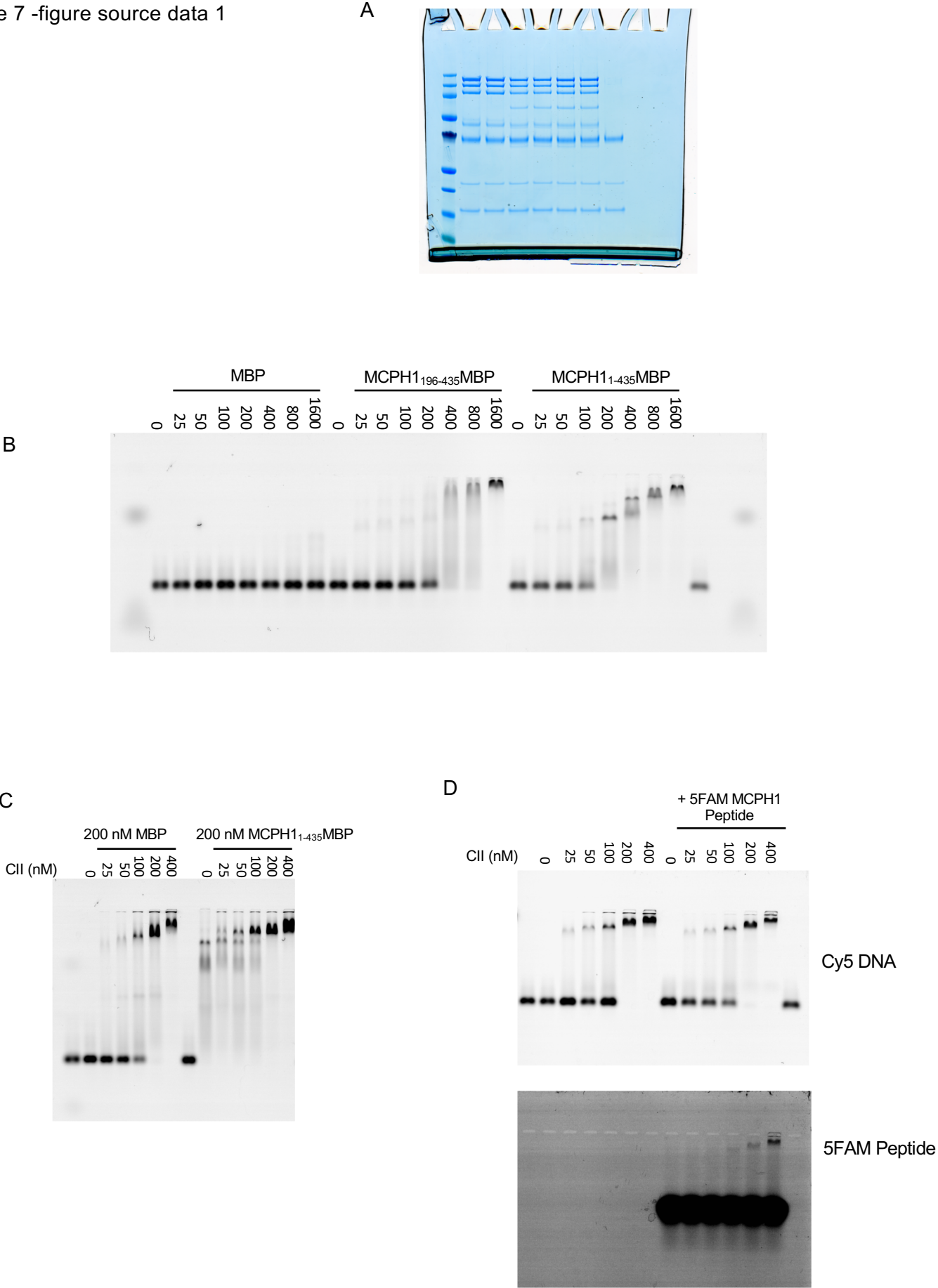

Supplement: Figure 7—source data 3. [file elife-73348-fig7-data3.zip › Figure 7-source data 3/Figure 7-source data 1.pdf]

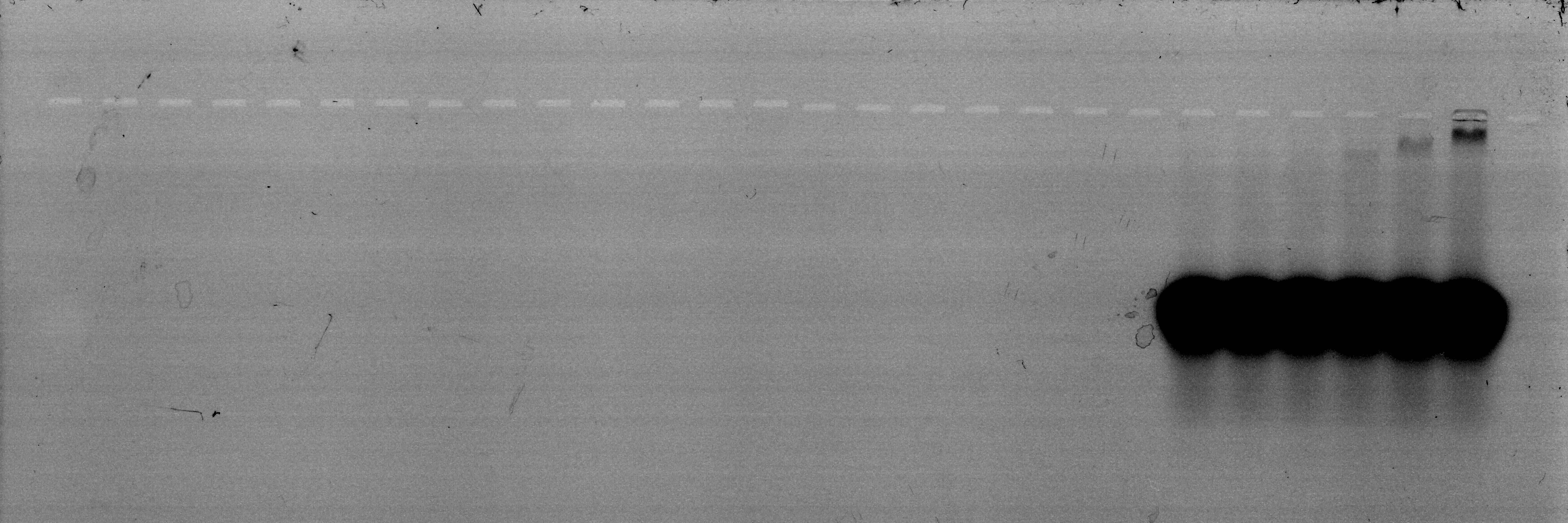

Supplement: Figure 7—source data 3. [file elife-73348-fig7-data3.zip › Figure 7-source data 3/Figure7D-source data 2.tif]

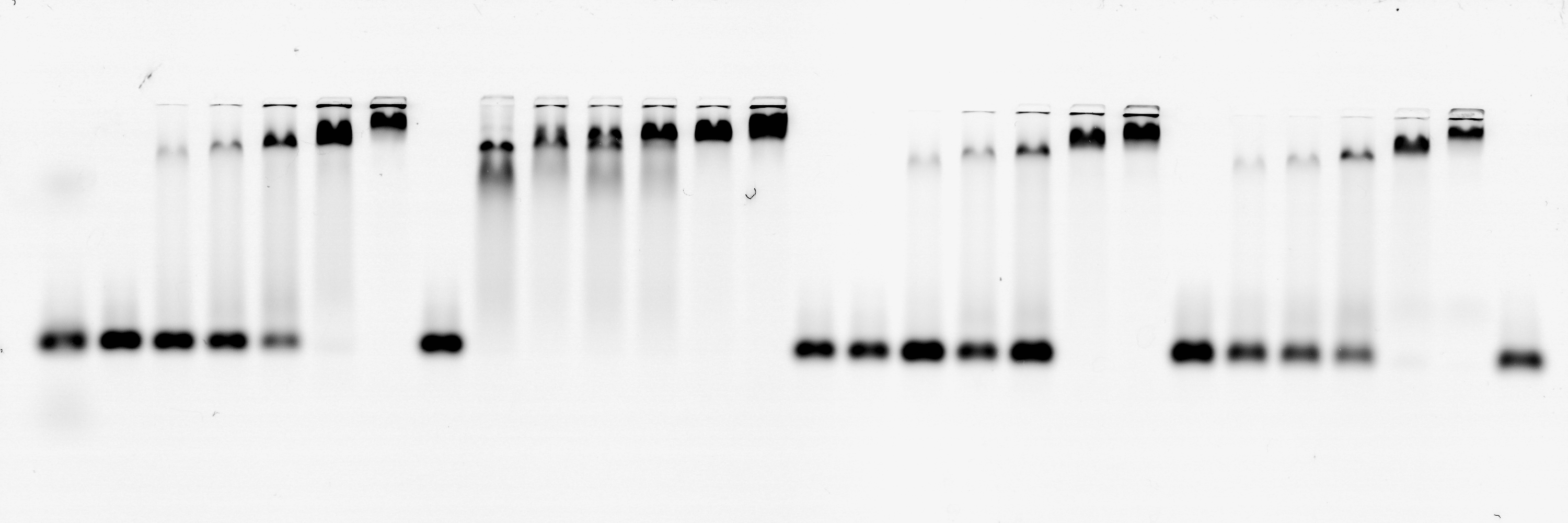

Supplement: Figure 7—source data 3. [file elife-73348-fig7-data3.zip › Figure 7-source data 3/Figure7D-source data 1.tif]

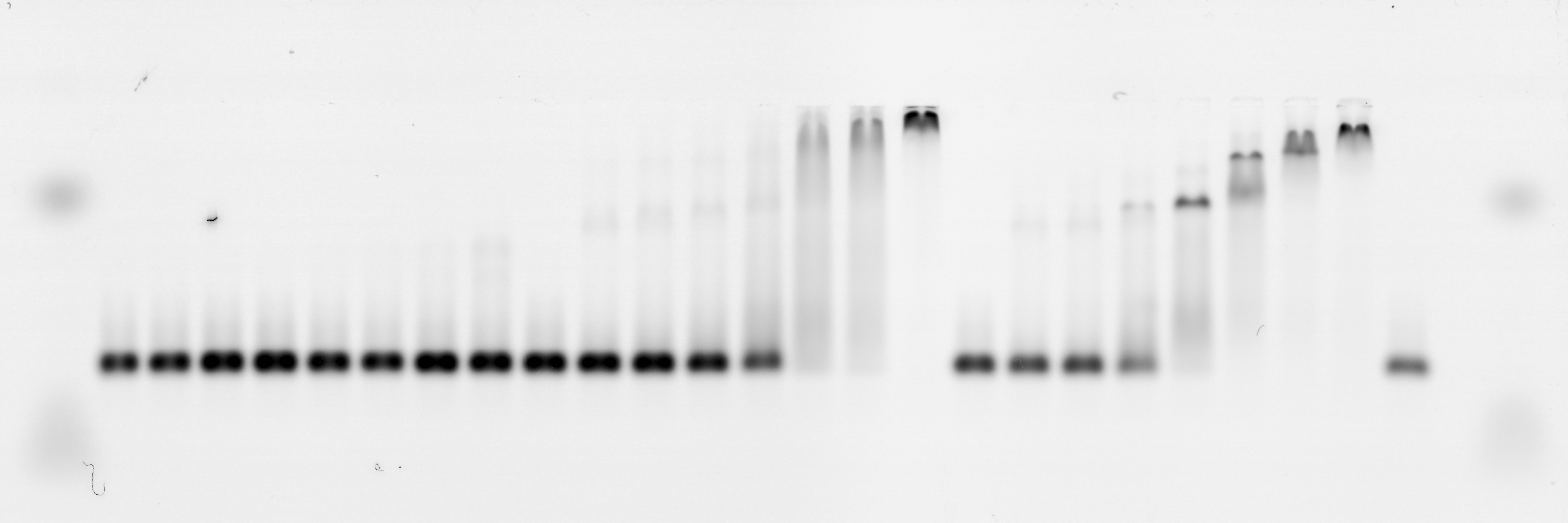

Supplement: Figure 7—source data 3. [file elife-73348-fig7-data3.zip › Figure 7-source data 3/Figure7B-source data 1.tif]

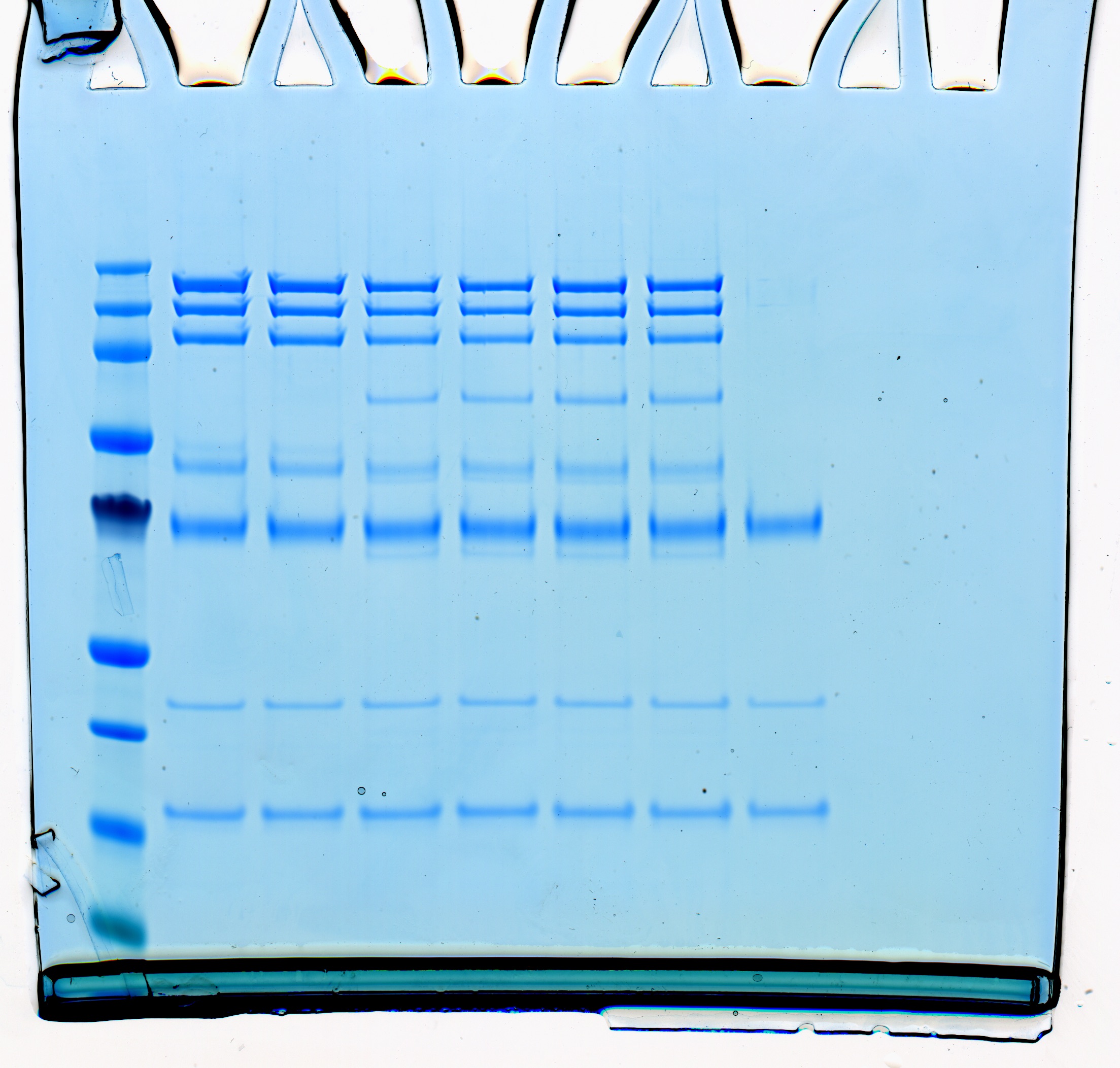

Supplement: Figure 7—source data 3. [file elife-73348-fig7-data3.zip › Figure 7-source data 3/Figure7A-source data 1.jpg]

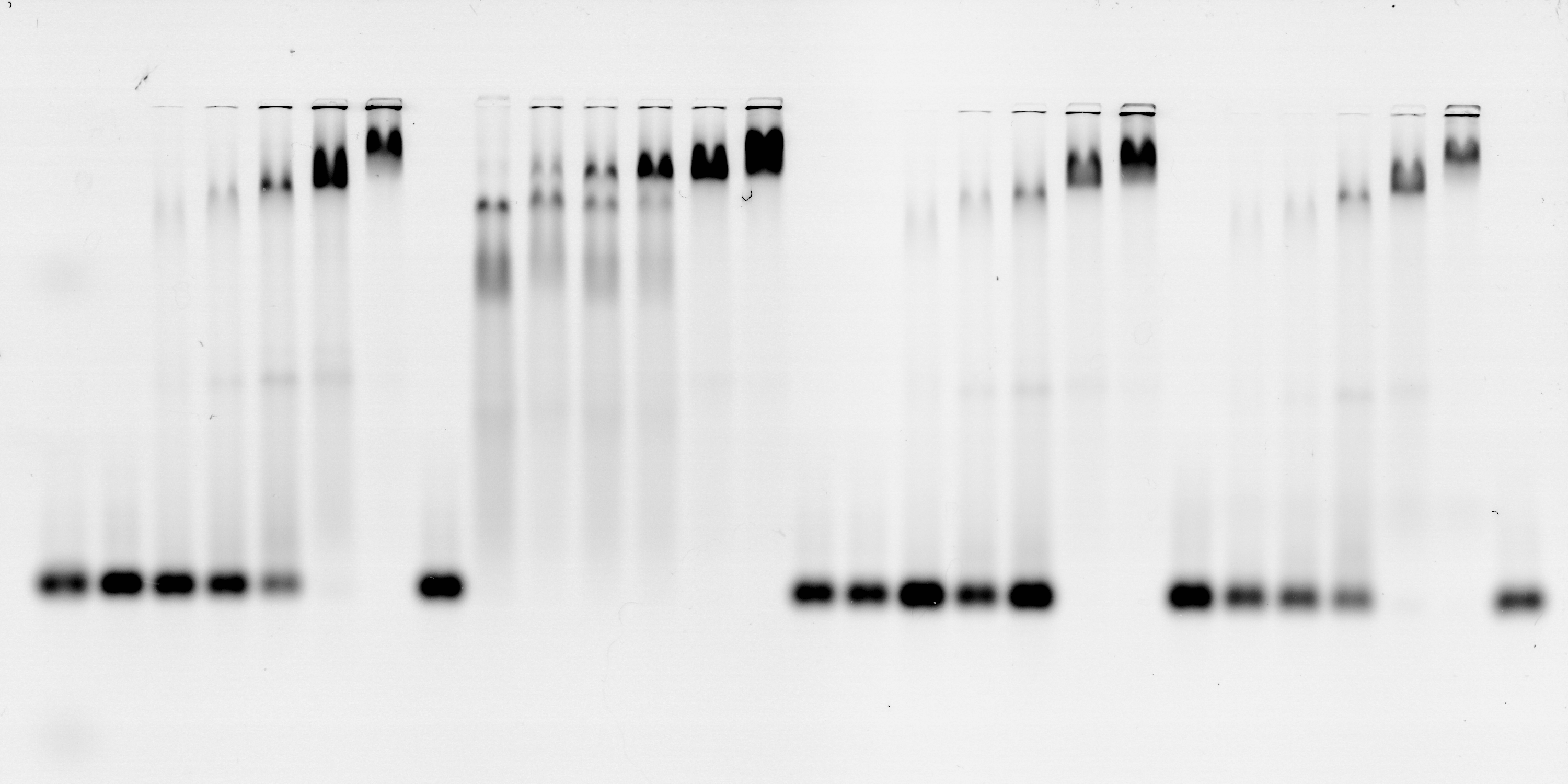

Supplement: Figure 7—source data 3. [file elife-73348-fig7-data3.zip › Figure 7-source data 3/Figure 7C-source data 1.tif]

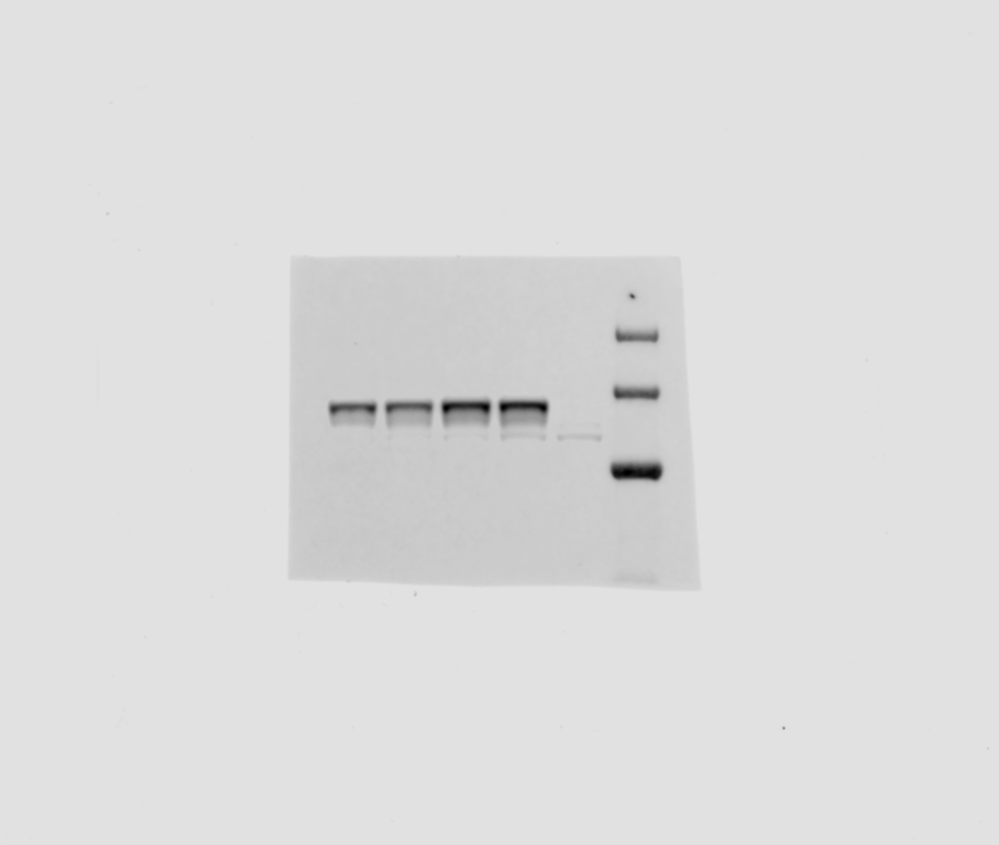

Supplement: Figure 10—figure supplement 1—source data 1. [file elife-73348-fig10-figsupp1-data1.zip › Figure 10-figure supplement 1-source data 1/Figure 10 -figure supplement 1-source data 1-anti-GFP.tif]

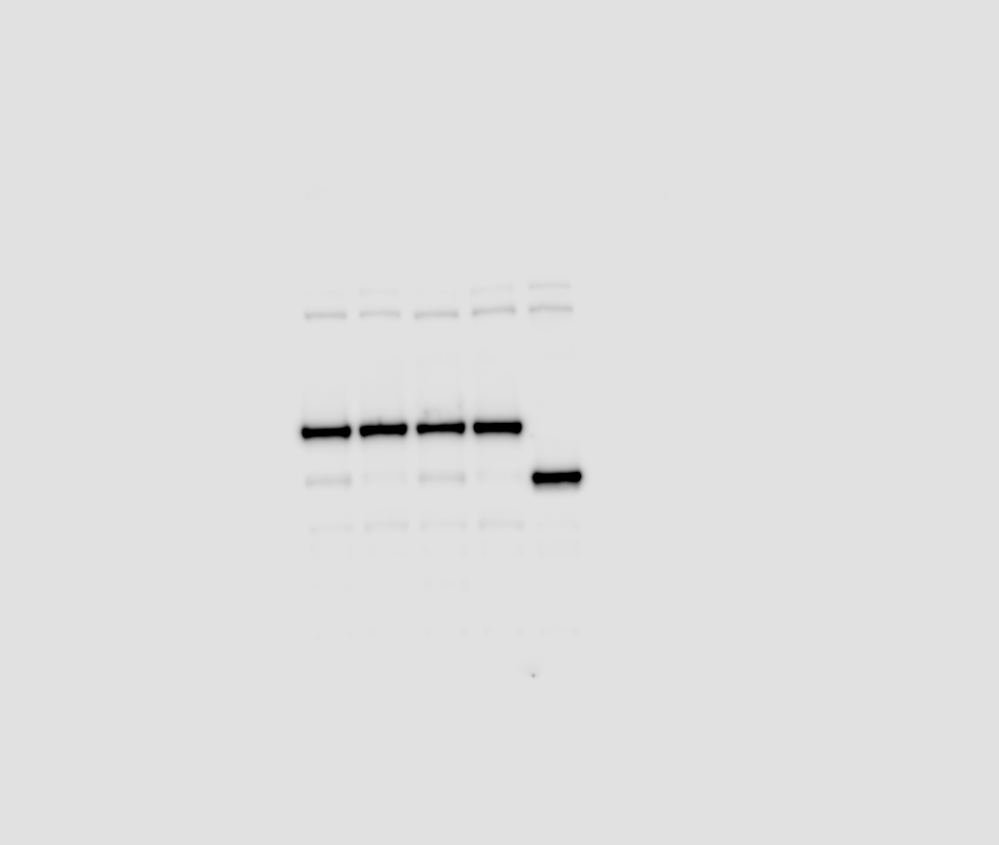

Supplement: Figure 10—figure supplement 1—source data 1. [file elife-73348-fig10-figsupp1-data1.zip › Figure 10-figure supplement 1-source data 1/Figure 10 -figure supplement 1-source data 1-anti Scc1.tif]

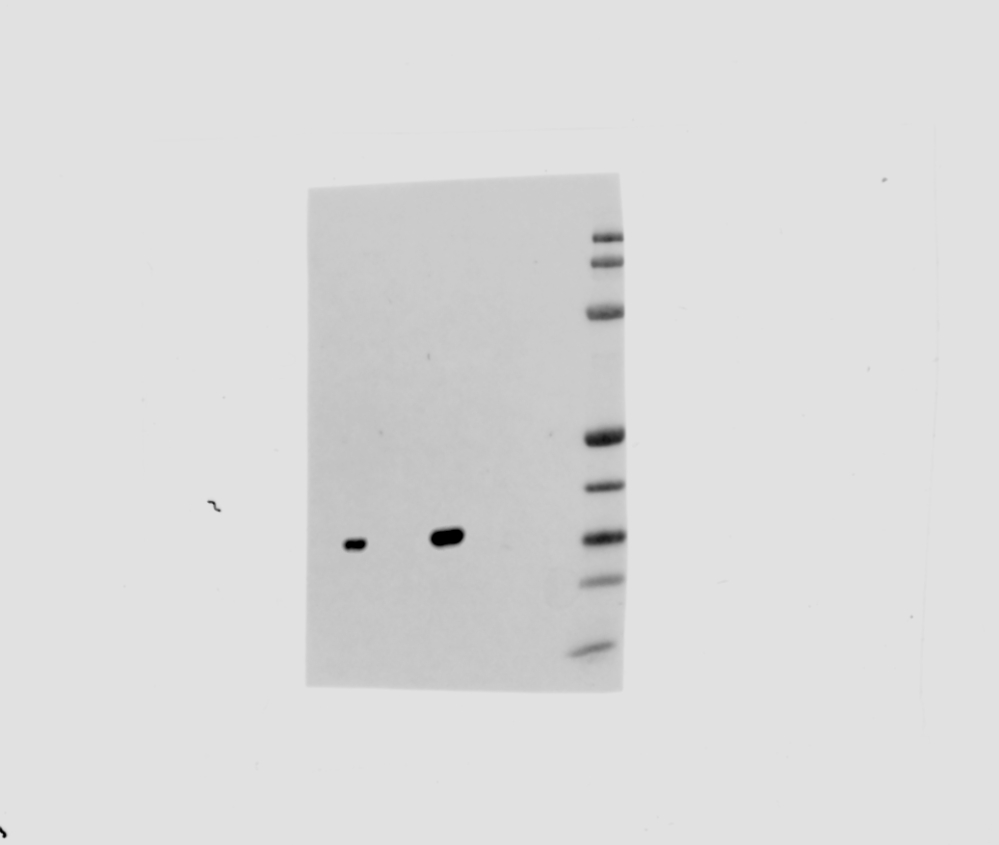

Supplement: Figure 10—figure supplement 1—source data 1. [file elife-73348-fig10-figsupp1-data1.zip › Figure 10-figure supplement 1-source data 1/Figure 10 -figure supplement 1-source data 1-anti V5.jpg]

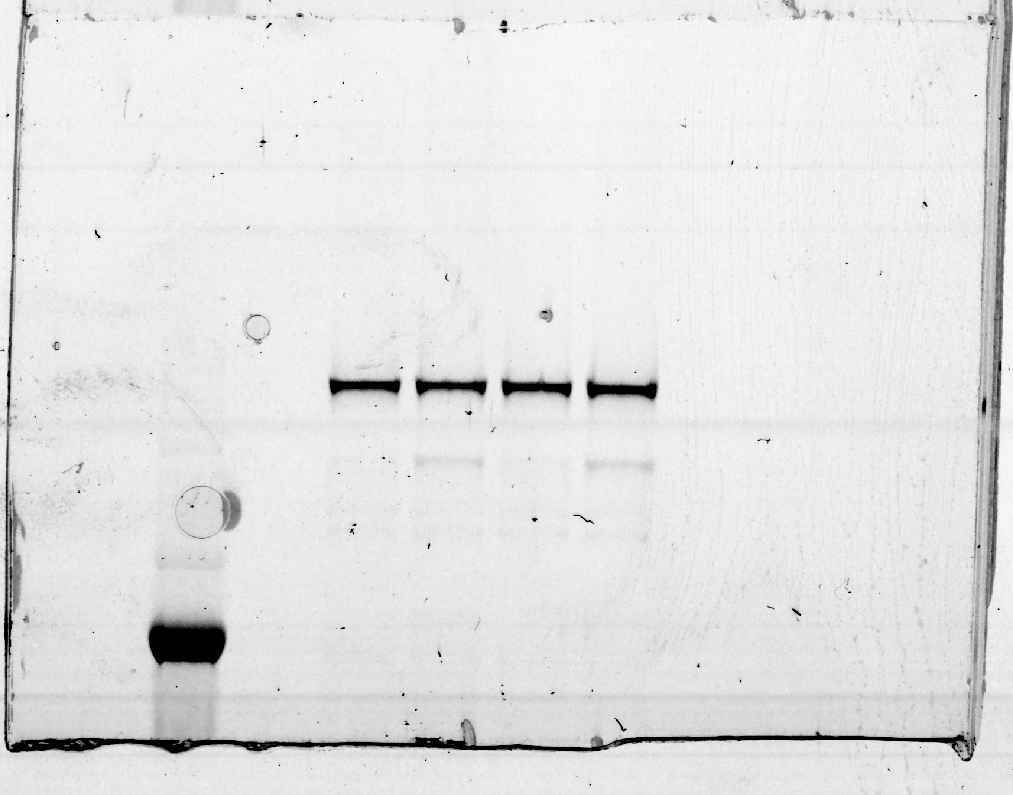

Supplement: Figure 10—figure supplement 1—source data 1. [file elife-73348-fig10-figsupp1-data1.zip › Figure 10-figure supplement 1-source data 1/Figure 10 -figure supplement 1-source data 1-SCC1 Halo TMR.tiff]

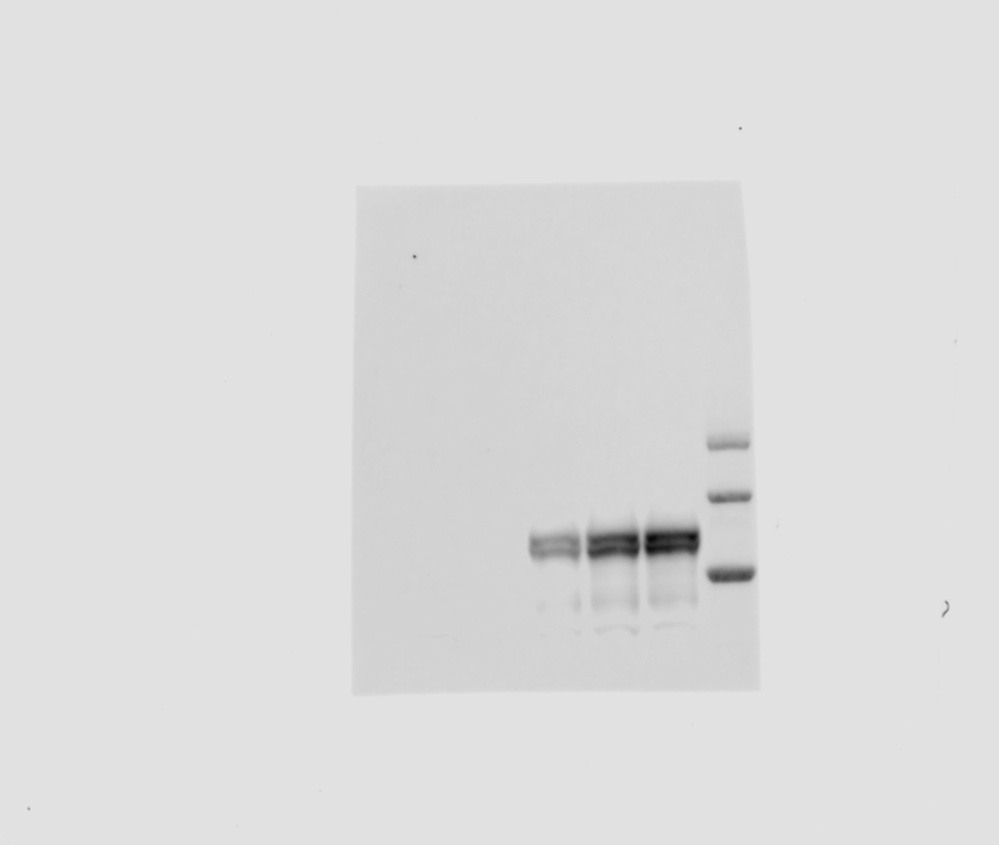

Supplement: Figure 10—figure supplement 1—source data 1. [file elife-73348-fig10-figsupp1-data1.zip › Figure 10-figure supplement 1-source data 1/Figure 10 -figure supplement 1-source data 1-anti MCPH1.jpg]

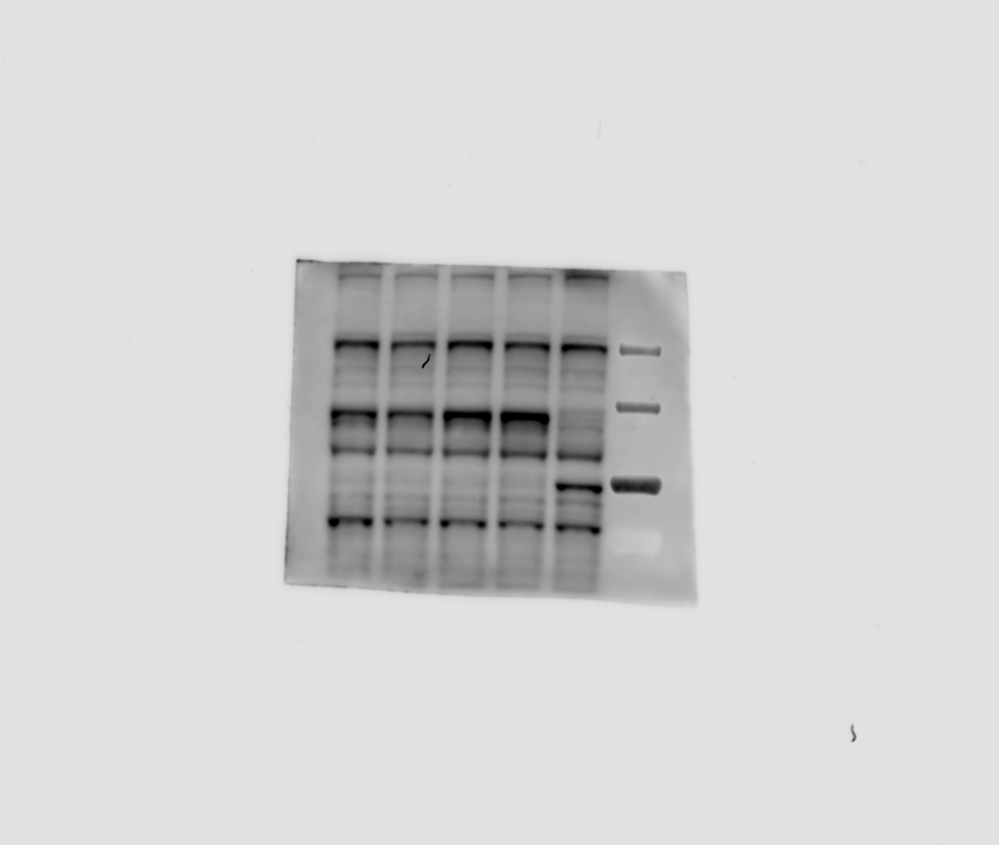

Supplement: Figure 10—figure supplement 1—source data 1. [file elife-73348-fig10-figsupp1-data1.zip › Figure 10-figure supplement 1-source data 1/Figure 10 -figure supplement 1-source data 1-antiNCAPH2.tif]

Figure 10 – figure supplement 1\_source data 1

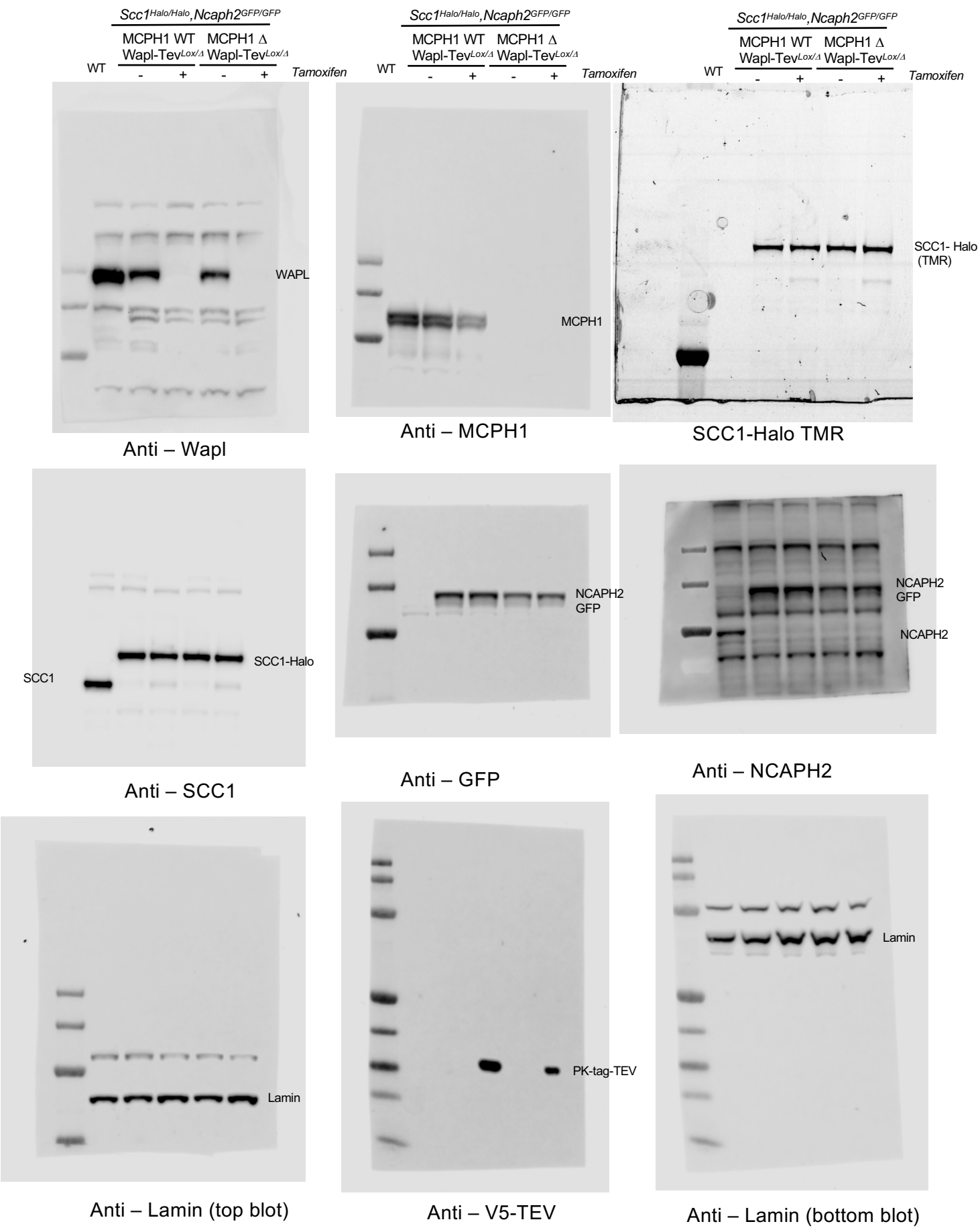

Supplement: Figure 10—figure supplement 1—source data 1. [file elife-73348-fig10-figsupp1-data1.zip › Figure 10-figure supplement 1-source data 1/Figure 10 -figure supplement 1-source data 1.pdf]

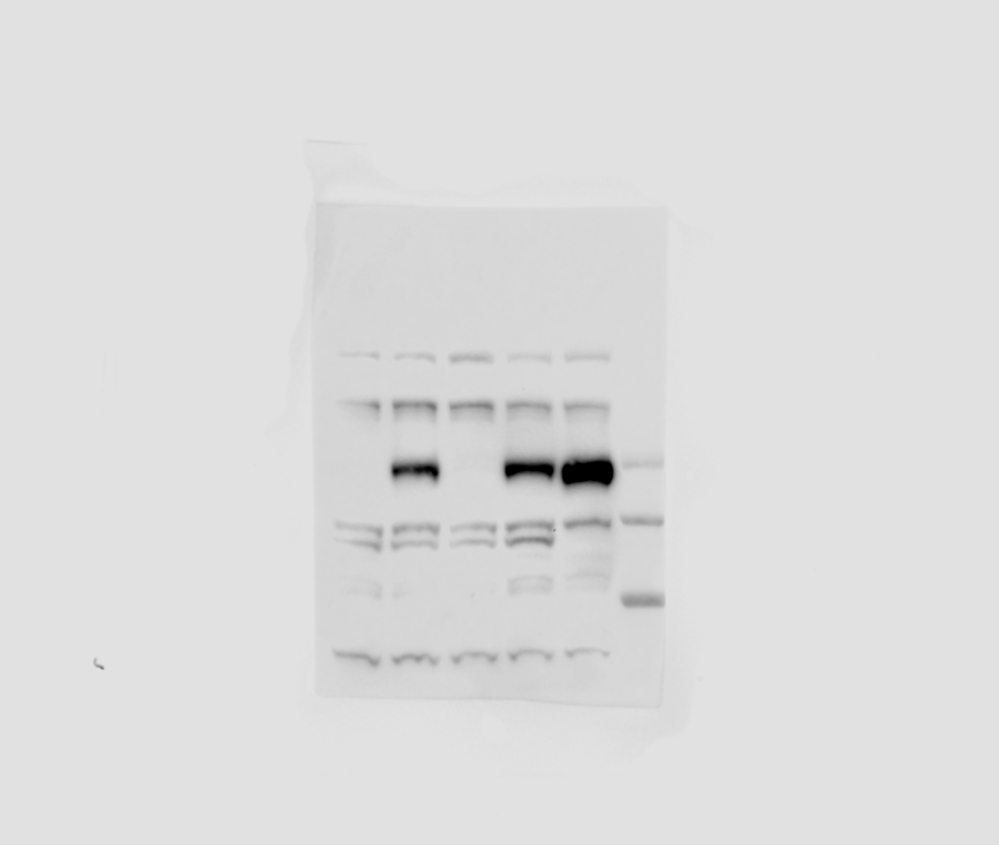

Supplement: Figure 10—figure supplement 1—source data 1. [file elife-73348-fig10-figsupp1-data1.zip › Figure 10-figure supplement 1-source data 1/Figure 10 -figure supplement 1-source data 1-anti Wapl.jpg]

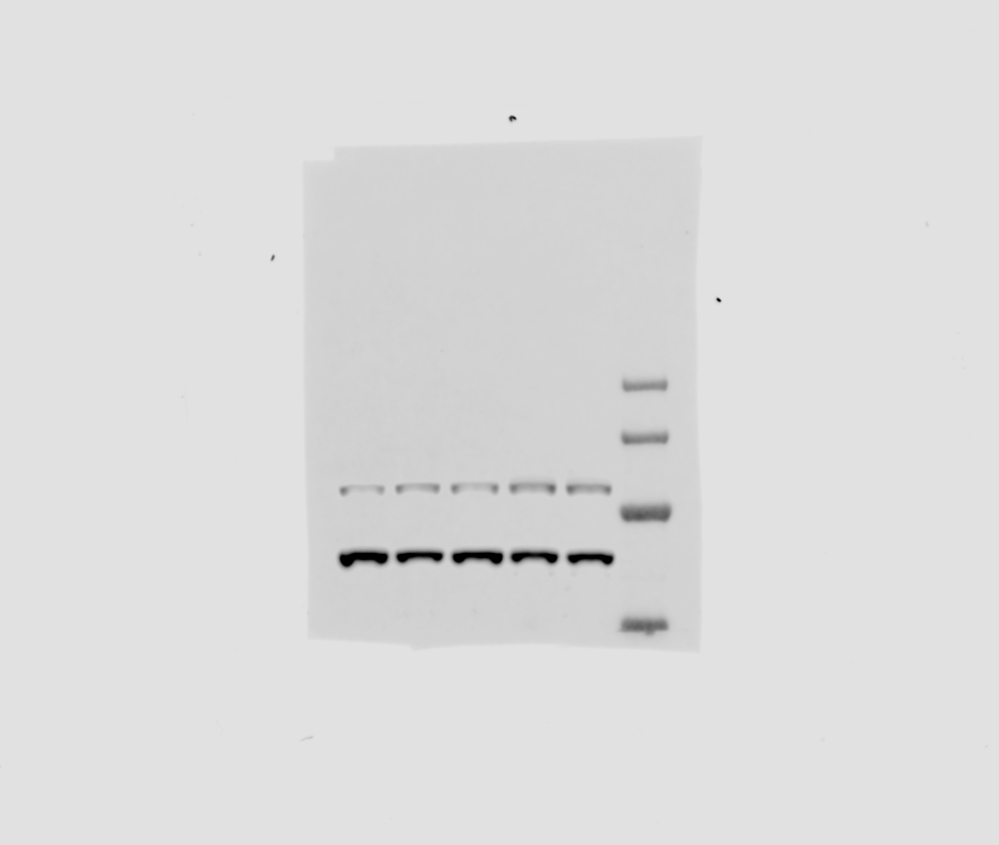

Supplement: Figure 10—figure supplement 1—source data 1. [file elife-73348-fig10-figsupp1-data1.zip › Figure 10-figure supplement 1-source data 1/Figure 10 -figure supplement 1-source data 1-anti Lamin 1.png]

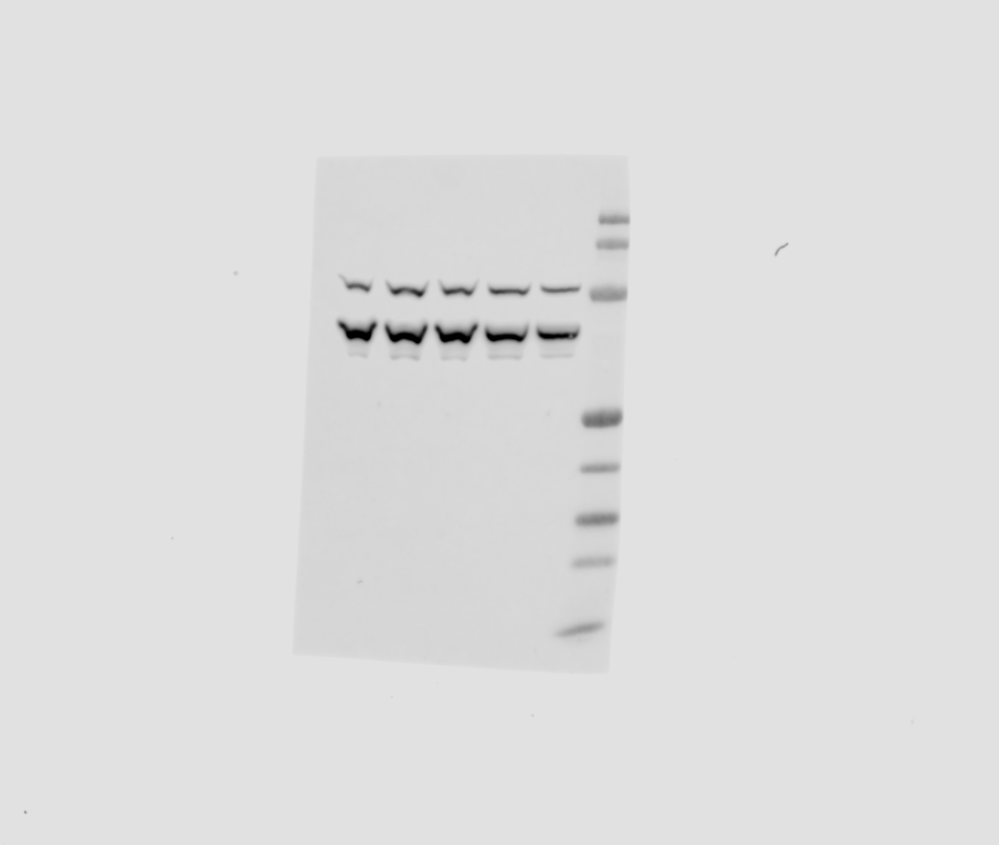

Supplement: Figure 10—figure supplement 1—source data 1. [file elife-73348-fig10-figsupp1-data1.zip › Figure 10-figure supplement 1-source data 1/Figure 10 -figure supplement 1-source data 1-anti Lamin 2.png]
